# Molecular modelling and simulation studies of the *Mycobacterium tuberculosis* multidrug efflux pump protein Rv1258c

**DOI:** 10.1371/journal.pone.0207605

**Published:** 2018-11-26

**Authors:** Ruben Cloete, Erika Kapp, Jacques Joubert, Alan Christoffels, Sarel F. Malan

**Affiliations:** 1 South African Medical Research Council Bioinformatics Unit, South African National Bioinformatics Institute, University of the Western Cape, Bellville, South Africa; 2 School of Pharmacy, University of the Western Cape, Bellville, South Africa; Universidade Nova de Lisboa Instituto de Tecnologia Quimica e Biologica, PORTUGAL

## Abstract

Mycobacterial efflux pumps play a major role in the emergence of antimycobacterial drug resistance. Of particular interest is the proteinaceous multi-drug efflux pump protein Rv1258c that encodes a tetracycline/aminoglycoside resistance (TAP-2)-like efflux pump which is active in susceptible and drug resistant *Mycobacterium tuberculosis*. Rv1258c is implicated in drug resistance to numerous antimycobacterials including first line drugs rifampicin and isoniazid as well as fluoroquinolone and aminoglycoside antibiotic classes. To date, compounds like verapamil and piperine have been shown to inhibit Rv1258c but no direct evidence for binding or mode of action exist. Therefore in the present study we generated an accurate 3D model of Rv1258c using MODELLER and validated its structure using molecular dynamic simulation studies with GROMACS software. The 3D-structures of Rv1258c and the homologous template 1pw4 were simulated within a POPE/POPG lipid bilayer and found to behave similar. Another important finding was the identification of one local energy minima state of the apo protein, which speaks to the flexibility of the protein and will be investigated further. Extraction of one of the open channel conformations of Rv1258c and blind docking of various structurally diverse putative inhibitors and substrates, allowed for the identification of a probable binding site. Spectinamide was found to bind to a different location on the outside surface of the protein suggesting its ability to avoid the efflux channel. We further identified 246 putative compounds that showed higher binding affinity values to Rv1258c compared to piperine and verapamil. Interaction analysis of the top 20 purchasable compounds identified crucial hydrogen bond interactions with Ser26, Ser45 and Glu243 as well as a pi-pi stacking interaction with Trp32 that accounted for the strong affinity of these compounds for Rv1258c. Future studies will entail purchasing a number of compounds for *in vitro* activity testing against *Mycobacterium tuberculosis*.

## Introduction

*Mycobacterium tuberculosis* is an obligate parasite and the causative agent of tuberculosis. It is an infectious disease that accounts for approximately 1.4 million deaths and 10.4 million new cases per year [1]. Currently, 6.3 million new cases of tuberculosis (TB) are diagnosed annually and today it still remains one of the most common causes of morbidity and mortality among human populations [1]. Despite the use of the Bacillus Calmette-Guérin (BCG) vaccine and effective antibiotics, the bacterium continues to thrive and has developed innate mechanisms to overcome drug treatment. The mechanisms by which *M*. *tuberculosis* develops resistance to tuberculosis drugs has been the focus of intensive research but it is recognised that efflux pumps play an important role in intrinsic as well as acquired genetic resistance [2–4].

Sequencing of numerous *M*. *tuber*culosis isolates has led to the identification of a number of open reading frames that encode putative efflux pumps. However, the exact mechanism of these pumps associated with intrinsic and acquired drug resistance has not been elucidated [5, 6]. One such efflux pump is Rv1258c, a proteinaceous active transporter localized in the cytoplasmic membrane of the cells that encodes a tetracycline/aminoglycoside resistance (TAP-2)-like efflux pump. [7, 8]. Previous studies have shown that deletion of Rv1258c from the *Mycobacterium bovis* BCG increases the susceptibility of the organism to isoniazid and rifampicin [9]. Additionally, Rv1258c plays an important role in multidrug-resistant (MDR) TB; and over-expression of Rv1258c, under rifampicin pressure, has been linked to increased transcription levels of this tap-like pump preventing cytosolic accumulation of drugs [8, 10]. This efflux pump has also been linked to resistance development to second line fluoroquinolone and aminoglycoside antibiotics [11].

Previous experimental studies showed that compounds like piperine, verapamil and chlorpromazine, inhibit the functioning of Rv1258c however no direct evidence have been provided for the binding and mode of action of these small molecules [3, 12, 13, 14]. Spectinomycin acts as a substrate while spectinamide avoids efflux through structural modification [15]. One study attempted the modelling of Rv1258c protein structure but did not use a template structure which is structurally similar (i.e shares a similar fold) to protein Rv1258c [12].

In the present study, we remodelled the structure of Rv1258c using a homologous protein that shares a similar fold and performed molecular dynamic simulation studies of the protein in a POPE-POPG lipid bilayer. We also investigated the possible binding of piperine, verapamil, chlorpromazine, spectinamide and spectinamycin to Rv1258c and describe their interactions with the efflux pump. In addition to this we identified novel compounds that could be used to inhibit Rv1258c utilising *in silico* pharmacophore modelling.

## Materials and methods

### Homology modelling

The PSIPRED web server was used to search for distantly related template structures for Rv1258c [16]. The amino acid sequence of Rv1258c was used as input and the option GenTHREADER was selected to search for templates [17]. The search yielded six templates that were categorized in the order of hit confidence based on highly significant p-value (less than 0.001) which suggest the correct secondary structure fold was assigned to the protein. We selected the top ranked template (PDBID: 1pw4) a crystal structure of the glycerol-3-phosphate transporter membrane protein family from *Eschericia Coli* based on an alignment confidence score of 331 (p = 4 x 10^−8^) which represents the most closely related structure to Rv1258c. The routinely used python scripts in MODELLER were used to perform an alignment prediction between the template (1pw4) and sequence Rv1258c using align2d.py [18, 19]. Thereafter, ten random 3D models were built for Rv1258c using the model-ligand.py script. The lowest discrete optimized protein energy (DOPE) score model (LDSM) was selected for further quality inspection as the lowest DOPE score model represents the more accurate protein model at its native conformation. The DOPE score profile for the model was plotted using Gnuplot and compared to the templates energy profile to identify regions of high energy [20]. The Ramachandran plot was used to interrogate the phi and psi dihedral angle distributions of C-alpha residues within the protein model using Procheck [21]. The Prosa-web server was used to calculate the normalised Z-score for the protein model and template to determine if the protein model predicted falls within range of high quality experimental structures with a similar size and shape [22]. The ERRAT overall quality factor and the 3D-1D compatibility score was calculated for the protein model and the template using the tools ERRAT and Verify3D located at (http://services.mbi.ucla.edu/SAVES/). Superimposing all atoms of the protein model onto the template provided an RMSD value, with lower values suggesting that there is very little deviation in main chain atoms and indicates that the correct fold was assigned to protein Rv1258c.

### Molecular dynamic simulation studies

The CHARMM-GUI interface was used to prepare protein-membrane systems for Rv1258c and template 1pw4 [23]. Briefly, we prepared two systems each; consisting of Rv1258c and 1pw4 in apo form. Each system was build using the membrane builder bilayer option with the protein aligned along the Z-axis of the membrane with the protein in the centre of the membrane (Z = 0). The number of lipids for the top (head) and bottom (tail) used for each system consisted of 96 palmitoyloleoylphosphatidylethanolamine (POPE) and 32 palmitoyloleoylphosphatidylglycerol (POPG) with a water thickness of 17.5Å from the top and bottom of the lipid head group and a per lipid hydration number of 50. We selected POPE and POPG as these are the main lipid components of the inner bacterial membrane [24]. Each system was neutralized by adding counter ions to each of the systems. These were 86 positive sodium and 28 negative chloride ions to apo 1pw4 to a 0.15 M concentration, while for the Rv1258c apo structure we added 91 potassium and 32 chloride ions. Each of the two systems underwent 50000 steps of steepest descents energy minimization to remove close van der Waals force contacts. Afterwards, all the systems were subjected to a two-step equilibration phase namely; NVT (constant number of particles, Volume and Temperature) for 500 ps to stabilize the temperature of the system and a short position restraint NPT (constant number of particles, Pressure and Temperature) for 500 ps to stabilize the pressure of the system by relaxing the system and keeping the protein restrained. The V-rescale temperature-coupling method was used for the NVT ensemble, with constant coupling of 0.1 ps at 300 K under a random sampling seed. For NPT, Parrinello-Rahman pressure coupling [25] was turned on with constant coupling of 0.1ps at 300K under conditions of position restraints (all-bonds). Electrostatic forces were calculated for both NVT and NPT using Particle Mesh Ewald method [26]. All systems were subjected to a full 200 ns simulation under conditions of no restraints.

The analysis of the trajectory files was done using GROMACS utilities. The root mean square deviation (RMSD) was calculated using g_rmsd and RMSD per-residue analysis using g_rms. The change in the solvent accessibility surface area (SASA) was calculated using g_sas to determine if the system reached convergence over the 200 ns simulation. Clustering analysis was performed using the GROMACS tool g_cluster to identify different conformational states of the protein. The Visual Molecular Dynamics package (VMD) was used to visually inspect motions along the trajectory [27]. Each 200 ns simulation was repeated at a random seed number to validate reproducibility of results.

### Principal component analysis and free energy landscape

Principal component analysis (PCA) is a statistical technique that reduces the complexity of a data set in order to extract biologically relevant movements of protein domains from irrelevant localized motions of atoms. For PCA analysis the translational and rotational movements were removed from the system using g_covar from GROMACS to construct a covariance matrix. Next the eigenvectors and eigenvalues were calculated by diagonalising the matrix. The eigenvectors that correspond to the largest eigenvalues are called "principal components", as they represent the largest-amplitude collective motions. We filtered the original trajectory and project out the part along the most important eigenvectors namely: vector 1 and 2 using g_anaeig from GROMACS utilities. Furthermore, we visualized the sampled conformations in the subspace along the first two eigenvectors using g_anaeig in a two-dimensional projection. Afterwards we oversimplified the calculation of the free energy landscape (FEL) using the GROMACS utility g_sham only along the first two eigenvectors. The FEL represents all the possible different conformations a protein can adopt during a simulation and are typically reported as Gibbs free energy. The molecules free energy was calculated with the formula ΔG(r) = − kBT ln P(x,y)/Pmin, where P is the probability distribution of the two Cartesian coordinates namely eigenvector 1 and 2, Pmin is the maximum probability density function, Kb is the Boltzmann constant and T is the temperature of the simulation. Conformations sampled during the simulation are projected on a two dimensional plane and the number of points in each cell was counted. The clustering of points in a specific cell represented a possible metastable conformation. All the simulations were carried out using the GROMACS 4.6.5 package [28] along with the CHARMM36 all-atom force field [29].

### Molecular docking

Putative inhibitors as well as likely substrates of the Rv1258c efflux pump were identified from literature and docked to Rv1258c using AutoDock Vina [30]. Blind docking was performed using Vina by setting the grid box over the whole protein to determine the binding site of verapamil, piperine, chlorpromazine, spectinamide, spectinomycin and BNG to Rv1258c as no experimental binding sites are known. Briefly, the receptor was prepared using AutoDock tools [31], which adds polar hydrogen’s, Gasteiger charges and saves the output file in pdbqt format, while the five small molecules were prepared with automated bash and python scripts namely, split_multi_mol2_file.py and prepare_ligand4.py. This was done to correct for errors such as missing atoms, added H_2_O, more than one molecule chain break and alternate locations. The various parameters for the docking process was stored in a configuration file named: conf.txt (S1 Text). The configuration file contained input parameters for the docking simulation such as: centre of mass coordinates, grid space and exhaustiveness of the search algorithm.

### Virtual screening

#### Pharmacophore search (ZincPharmer)

A pharmacophore is the chemical feature(s) essential for binding of a ligand to a receptor or enzyme, which accounts for its effect on the protein’s activity [32]. The single protein drug complexes of Rv1258c_verapamil and Rv1258c_piperine were used to generate a pharmacophoric model based on the interactions made by the docked small molecules. There were two features critical for binding and these were hydrogen-bonds and hydrophobic interactions, which were included in the pharmacophore model. Subsequently, the generated pharmacophore model was screened against the ZINC database to identify novel potential lead compounds that share similar chemical features when aligned in 3D space. The Pharmer technology implemented in ZINCPharmer is a “high-performance search engine that employs novel search methods such as; geometric hashing, generalized Hough transforms and Bloom fingerprints to perform an exact pharmacophore match and accelerate the search algorithm” [33]. The search yielded 548 compounds for the Rv1258c docked complexes when screening the ZINC database for purchasable compounds. These were further validated using docking studies.

#### Compound docking and scoring

The 548 compounds identified for the Rv1258c docked complexes from the ZINC database were prepared with automated bash and python scripts namely, split_multi_mol2_file.py and prepare_ligand4.py as mentioned previously. The same input parameters from the previous docking were used and the output of the docking run was filtered based on the docking score measured as kcal/mol. Subsequently, the interactions of the top binders that showed higher binding affinity than the five putative inhibitors was analysed using Poseview [34]. Poseview calculates four types of interactions namely; I) hydrogen bonds, II) hydrophobic, III) metal interactions and IV) π interactions.

## Results

### Molecular modelling and quality assessment

**Fig 1:**
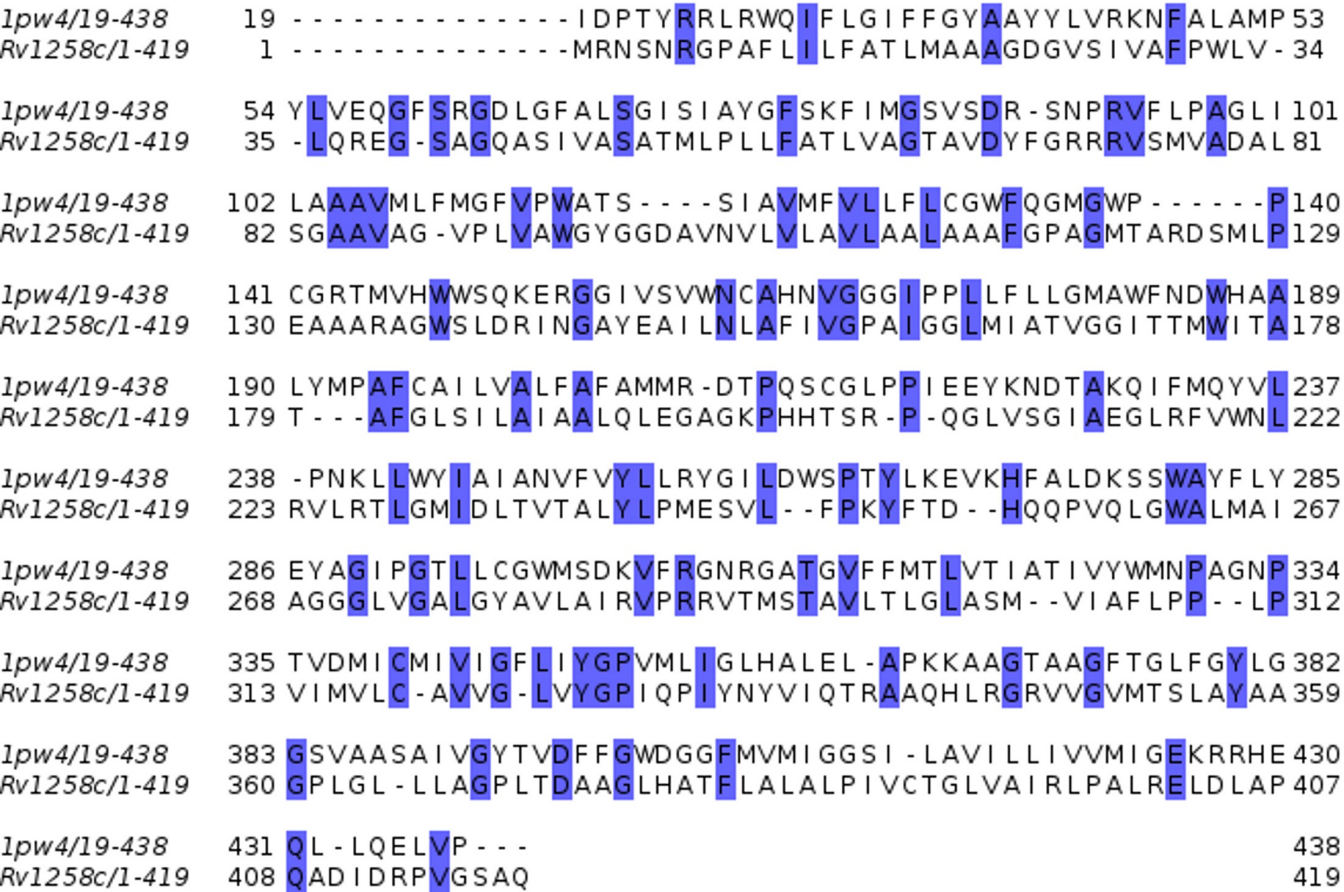
Sequence to structural alignment of Rv1258c to homologous template 1pw4. Blue highlighted boxes show conserved residues.

**Fig 2:**
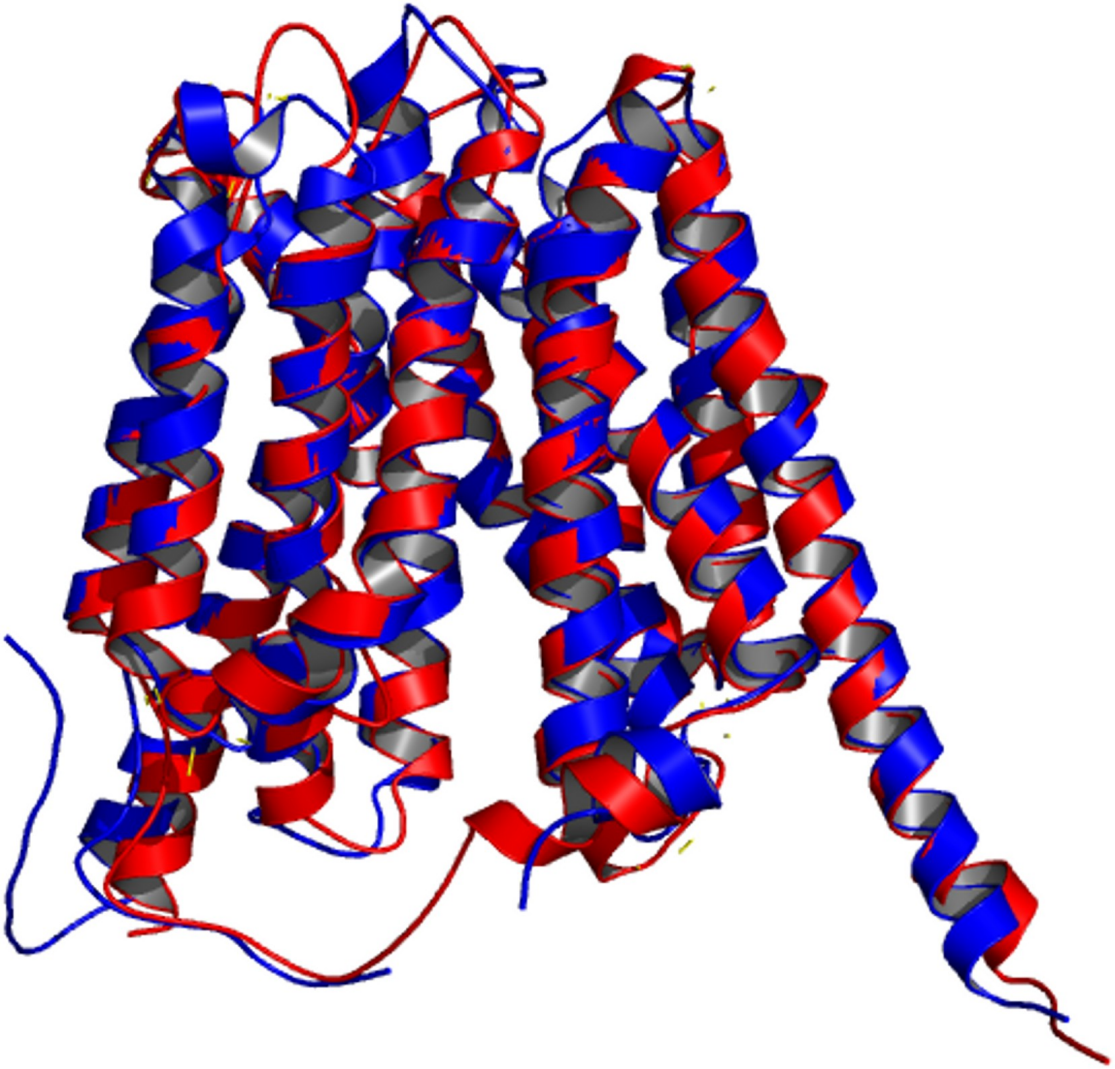
Superimposition of the LDSM Rv1258c (red) on the homologous template 1pw4 (blue).

The amino acid sequence of Rv1258c shared very low sequence identity of approximately 24% and an overall sequence coverage of 96% to that of the template 1pw4 (Fig 1). The overall arrangement of the secondary structure for the LDSM of Rv1258c is similar to that of template 1pw4 consisting of 17 helices, one beta sheet and 18 coil regions (S1 Fig). Although the sequence identity is low, the RMSD value after superimposition and aligning all atoms of the predicted model to the template 1pw4 was found to be 0.720 Å (Fig 2). This suggests very little deviation between carbon main chain atoms and indicates homology between the two structures. We also performed sequence and structural comparisons between the amino acid sequences of Rv1258c, Sharma (2010) template (2GFP) and 1pw4 and 3D structures of the three proteins using BLASTp pairwise alignment and Pymol. The sequence identity was found to be 36% between Rv1258c and template sequence 2GFP and 24% with 1pw4. Comparing the sequence identity between template 1pw4 and 2GFP indicated a score of 26%, suggesting the two proteins are not homologs. Although, the sequence identity was low between the template 1pw4 and Rv1258c the fold was more similar to our predicted model for Rv1258c. The structural similarity between our predicted model and the template 2GFP was 7.16Å suggesting low similarity between the two 3D structures because the structural deviation was more than the thresh-hold value of 2 Å (S2 Fig). Furthermore, aligning template 1pw4 used for our model to template (2GFP) showed an RMSD of 10.37 Å again suggesting large structural deviation between the two templates (S3 Fig). Additional inspection of the DOPE score energy profiles indicated no regions of extremely high energy, suggesting that the model is a reliable approximation of the protein’s native fold (S4 Fig). The Prosa Z-score for the LDSM was equal to -4.57, comparable to the template Z-score of -5.29, suggesting that the model falls in the range of medium quality crystal structures of a similar size and shape. Ramachandran plot analysis indicated that more than 90% of residues for the LDSM of Rv1258c were found in favoured regions, meaning that 90% of residues in the protein structure satisfied phi and psi dihedral angle distributions. The ERRAT overall quality factor was calculated to be 72.66 and 73.71 for the LDSM and the template, respectively, which is above the expected accuracy tress-hold of 70% for medium resolution structures considering the template had a resolution value of 3.3 Å. Furthermore, using Verify3D we found that 63.25% of the model residues had an averaged 3D-1D score > = 0.2 while for the template 1pw4, 65.90% of the residues had an averaged 3D-1D score > = 0.2, suggesting similarity between the two structures although both failing the 3D profile validation test. This is expected as the sequence identity is fairly low between the target and the template. Overall, the predicted model satisfied (RMSD, Prosa Z-score, ERRAT and Procheck) quality evaluation checks.

### Molecular dynamic simulation

**Fig 3:**
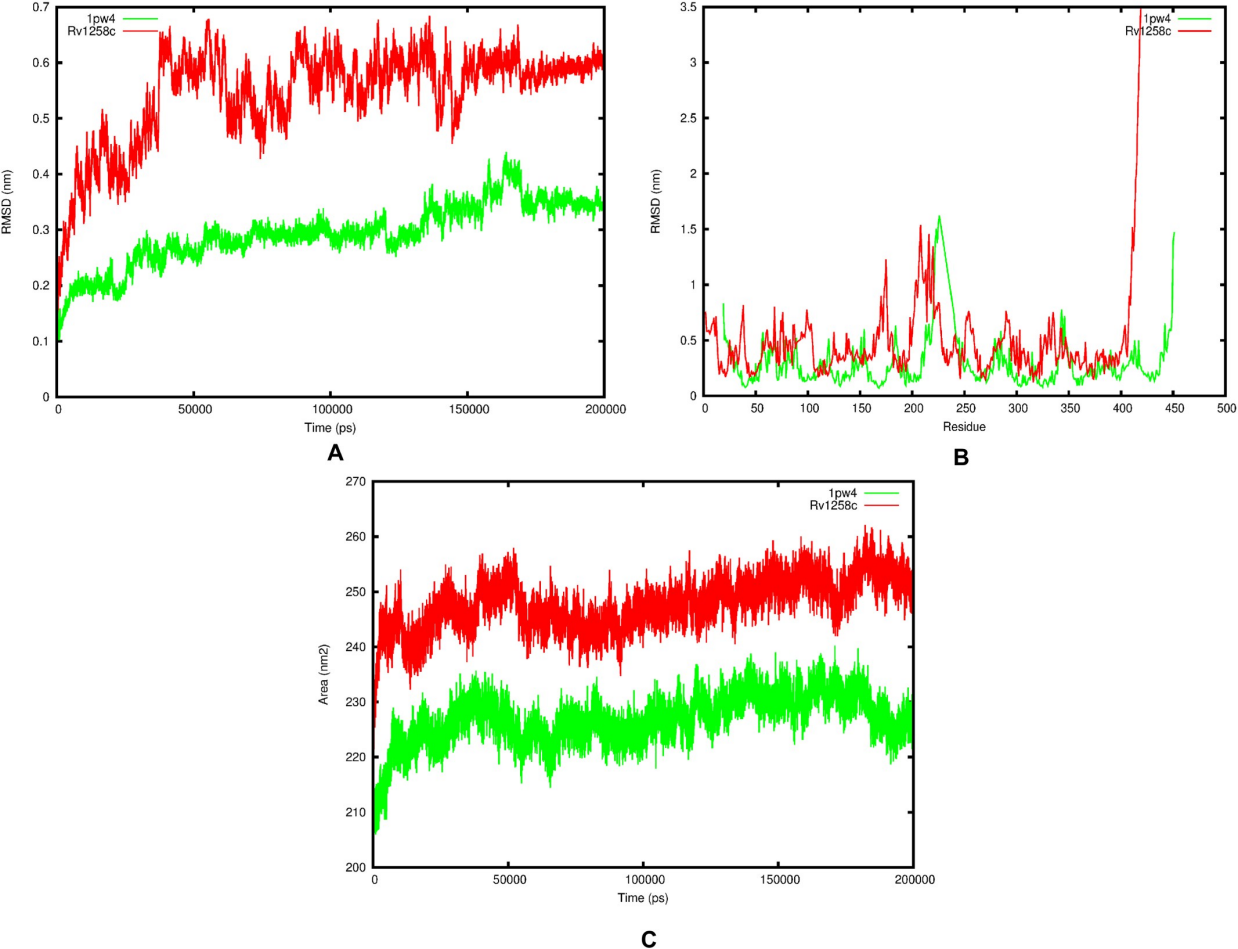
Trajectory analysis for Rv1258c (red) and 1pw4 (green) over the 200 ns simulation. (A) RMSD analysis of the backbone atoms, (B) RMSD per-residue analysis of the protein atoms and (C) The total SASA values of the protein atoms.

**Fig 4:**
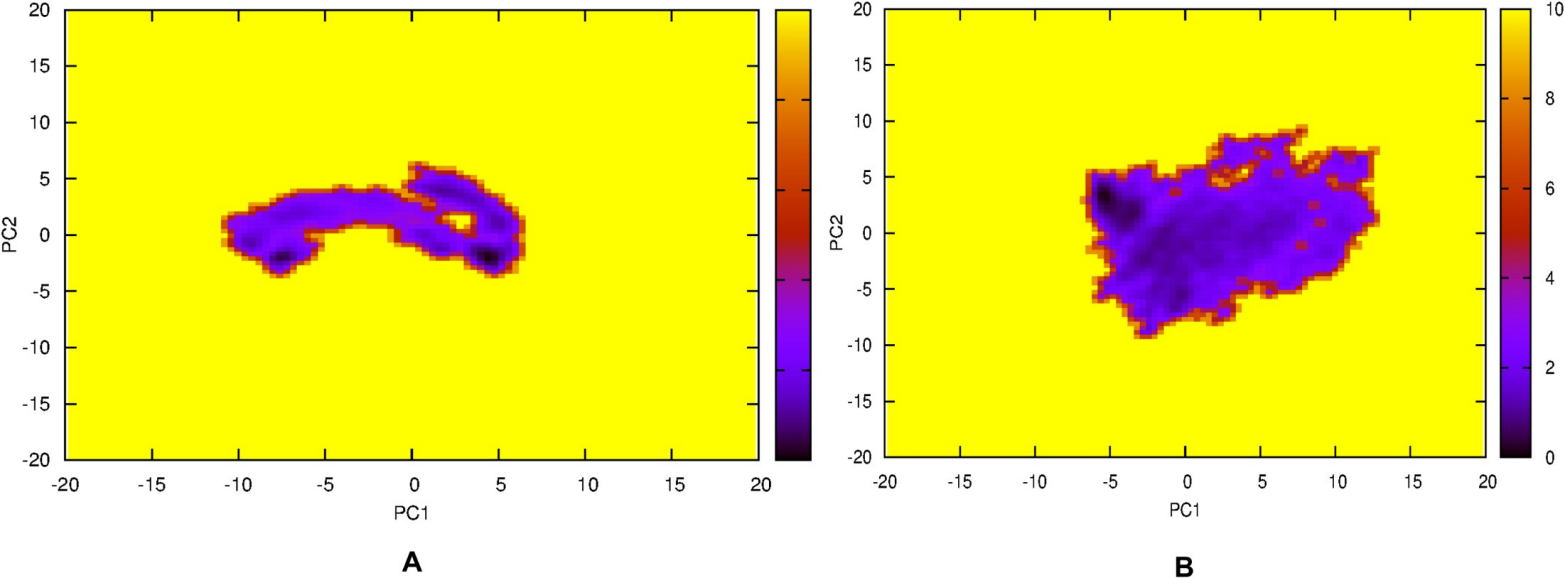
Free energy landscape of the protein backbone atoms for principal components 1 and 2 over 200 ns simulation. (A) For Rv1258c and (B) For 1pw4.

The RMSD analysis of backbone atoms for the two systems confirmed similar behaviour whereby both Rv1258c and 1pw4 sampled both open and closed conformational states during the 200 ns simulation (Fig 3A), although the average mean for 1pw4 of (0.29 ±0.06 nm) suggest it is more stable than Rv1258c (0.55 ±0.09 nm). This is visually illustrated in the two simulation movies (S1 and S2 Movies). The RMSD per-residue analysis of the two systems indicated that 1pw4 had lower RMSD values of approximately 0.28 ±0.21 nm compared to 0.48 ±0.36 nm for Rv1258c, and with only one region encompassing residues (200–250) with extremely high fluctuation for 1pw4, but this corresponded to a C-terminal loop regions that contained a breakage in that region. Additionally, this region does not contain the known substrate binding site residues R45 and R269 (Fig 3B). Furthermore, the RSMD per-residue analysis indicated two regions (170–176, 200–230) with high RMSD fluctuation values that corresponded to flexible loop regions within the protein (Fig 3B). The total SASA values for the two proteins showed similar fluctuation patterns and reached equilibrium (Fig 3C). Again the total SASA values were lower for 1pw4 (227 ±4.57 nm^2^) in comparison to Rv1258c (248 ±4.71 nm^2^). Principal component analysis of the first two eigenvectors for the two trajectories indicated smaller randomised movements for 1pw4 compared to Rv1258c after diagonalisation of the covariance matrix (a square matrix of numbers that describe the directionality of the eigenvector) with values 51.07 nm and 60.81 nm, respectively. Calculating the contribution of each PCA component for each system indicated for 1pw4 PCA component 1 and 2 each contributed 63 and 11% of the overall motion while for Rv1258c PCA components 1, 2, 3 and 4 each contributed 37, 20, 15 and 9%, respectively. Plotting the movement of the first two principal components throughout the free energy landscape indicated two metastable clusters for 1pw4, while Rv1258c showed only one conformational cluster (Fig 4A and 4B). Clustering analysis of the Rv1258c trajectory yielded 7 clusters at an 0.35 nm average RMSD cut-off value. We selected one representative structure from the cluster that corresponded to time point (t = 190700ps) and that represented a metastable conformation as identified from the PCA analysis for docking studies.

### Docking and interaction analysis

Autodock VINA was first used to perform a convergence test that included blind docking of six small molecules to the open channel conformational cluster 7 of Rv1258c at exhaustive parameter values of 16, 32, 64 and 128. Afterwards, docking was performed at an exhaustive value of 50 to determine which of the six small molecules showed the highest affinity for the Rv1258c enzyme. The six small molecules were first inspected to determine if the docking results showed convergence based on the clustering of the top predicted modes using Pymol. Based on the visual inspection clustering of the top five modes occurred more frequently at higher exhaustiveness values 64 and 128. This is reported for piperine and Verapamil as they obtained the highest binding affinity scores (S5–S12 Figs). The top modes also exploited the same binding space indicating that the search space was extensively explored by the algorithm. The docked molecules (piperine, spectinomycin, spectinamide, chlorpromazine and verapamil) to the Rv1258c cluster indicated that piperine had the strongest binding affinity (-9.3 kcal/mol) followed by verapamil (-8.4 kcal/mol) and then by chlorpromazine (-7.7 kcal/mol), spectinomycin (-7.6 kcal/mol) and spectinamide (-7.4 kcal/mol). Interestingly, spectinamide showed a higher binding affinity than spectinomycin although binding to an allosteric site located on the outside solvent accessible surface area of the protein. The sugar, BNG, showed the lowest binding affinity for Rv1258c (-6.2kcal/mol). To validate the binding affinity values we performed interaction analysis to identify the residues that contributed to the overall binding energy. Interaction analysis indicated that verapamil and piperine showed the highest number of hydrophobic interactions each having 5 non polar contacts followed by chlorpromazine, spectinomycin and BNG with each four hydrophobic contacts, while spectinamide showed no hydrophobic contacts (Table 1, S13–S18 Figs). Spectinamide showed the highest number of hydrogen bond interactions (3), compared to spectinomycin, BNG and chlorpromazine having 2, 2 and 1 polar contact with Rv1258c, respectively (Table 1, S13–S18 Figs).

**Table 1:** Docking scores and interaction residues for the five putative inhibitors and potential substrate BNG.

**Protein**	Cluster	Compound	Docking score(kcal/mol)	H-bonds	Hydrophobic	Pi-PiStacking
Rv1258c	7	Piperine	-9.3	None	5 (Trp32, Glu243, Phe247, Phe251, Gly363)	None
		Verapamil	-8.4	None	5 (Trp32, Gly161, Ile165, Pro248, PHE251)	None
		Chlopromazine	-7.7	1 (Glu243)	4 (Val28, Pro248, Phe251, Leu264)	None
		Spectinomycin	-7.6	2 (Val25, Glu243)	None	None
		Spectinamide_1599	-7.4	2 (Asp125, Arg217, Asn221)	None	None
		BNG	-6.2	2 (Thr179, Leu183)	4 (Leu33, Met164, Ile165, Val168)	None

BNG, B-nonylglucoside; H-bonds, hydrogen bonds, Pi-Pi aromatic stacking interaction.

The numbers in front of brackets indicate the total amount of interactions formed with the residues listed inside the brackets.

### Pharmacophore search and docking

The Rv1258c-verapamil and -piperine complexes were selected as a pharmacophore to search for potential compounds that could inhibit Rv1258c as these compounds demonstrated the lowest binding energy of the 5 compounds evaluated in the initial blind docking. Both verapamil and piperine has previously been shown to inhibit Rv1258c experimentally although the mechanism by which inhibition occurs is still under debate [3, 12, 13, 14, 15, 35]. Both these compounds also docked to the same binding pocket site on the protein model of Rv1258c and showed two similar interacting residues Trp32 and Phe251. The interactions observed from the previous docking experiment were used to generate pharmacophore features for each complex. The Rv1258c-verapamil complex had five hydrophobic and we included one hydrogen bond feature and Rv1258c-piperine had three hydrophobic and three additional hydrogen bond features and only one additional aromatic feature. Including more features increases the likelihood of finding compounds with higher specificity for Rv1258c. Using these features, the ZINC database was searched using ZINCPHARMER for potential compounds that shared similar features to the two small molecules aligned in three dimensional space. For cluster 7 we identified in total 548 hits for Rv1258c-piperine and Rv1258c-verapamil. Each of the compounds identified were validated by docking them to cluster 7 (open channel cluster) using VINA to determine if they showed higher binding affinity compared to that of verapamil and piperine. Out of the total 548 compounds 246 showed higher affinity than verapamil and piperine. Subsequently, interaction analysis of the top 20 ranking compounds provided support for the higher binding affinity which was due to important hydrogen-bond interactions between the compounds and residues Ser26, Ser45, Glu243 and a pi-pi stacking interaction with Trp32 of Rv1258c (Table 2, S19–S38 Figs). We also observed a variety of hydrophobic interactions being formed between the top 20 compounds and Rv1258c non polar residues ranging from one to seven (Table 2, S19–S38 Figs).

**Table 2:** Docking scores and interaction residues for the top 20 compounds bound to Rv1258c cluster 7.

Cluster	Docking score(kcal/mol)	ZINC_ID	Hydrogen-bond	Hydrophobic	Pi-Pi stacking
7	-11.2	ZINC36652490	Ser26	Ala29, Leu33, Val168	-None
	-11.0	ZINC16608089	Ser45	Val25, Val28	Trp32
	-11.0	ZINC35729457	Ser45, Glu243	Val25, Pro158	None
	-11.0	ZINC35729461	Ser45	Trp32, Leu33,Met164, Pro248	None
	-10.9	ZINC16608087	Ser45	Val25, Ile27, Val28, Ala29, Trp32, Leu264	Trp32
	-10.9	ZINC15947760	Glu243	Gly363	None
	-10.9	ZINC90733545	Ser45	Trp32, Leu33, Met164Pro248	None
	-10.8	ZINC45943747	Ser45	Val28, Ala29, Trp32,Pro248,	None
	-10.8	ZINC08920371	Glu243	Val25, Trp32,Ala44, Ala48, Pro248,	None
	-10.8	ZINC72034391	Ser45	Leu240	None
	-10.7	ZINC45943751	Ser45	Val28, Ala29, Leu33,Met164, Pro248, Leu264	None
	-10.7	ZINC16608126	Ser45	Val25, Leu264	Trp32
	-10.7	ZINC08920366	Glu234	Val25, Trp32, Ala44, Pro248	None
	-10.7	ZINC06793104	None	Trp32, Ala41, Ala44, Pro158, Leu246, Pro248, Leu264	Trp32
	-10.7	ZINC45943745	Ser45	Val 29, Ala29, Trp32, Ile165, Pro248	None
	-10.6	ZINC09701714	Ser26	Leu33, Met164, Val168, Leu264	None
	-10.6	ZINC35933009	Ser45	Val25, Ala29, Trp32, Pro248, Leu264	None
	-10.6	ZINC78721136	Trp32	Val28, Ala29, Trp32, Leu33,Met164, Leu264	None
	-10.6	ZINC15947762	Glu243	Gly363	None
	-10.3	ZINC16608150	Ser26	Val28, Ala29, Trp32, Leu33, Pro248	Trp32

## Discussion

The mechanism of efflux pump inhibition of piperine and verapamil and other efflux pump inhibitors has been under debate for some time. The absence of a crystal structure for Rv1258c made it difficult to prove (or disprove) inhibition of efflux function through direct binding to the protein. Particularly in the case of verapamil, a number of studies propose conflicting mechanisms for observed augmented antimycobacterial efficacy in *Mycobacterium tuberculosis* and the possibility exists that this may be due to a multimodal mechanism of action [13, 14, 35, 36]. Verapamil is a calcium channel blocker and has been shown to have a significant inhibitory effect on mycobacterial efflux pump activity but has severe adverse effects at high concentrations and therefore, cannot be considered for tuberculosis treatment in humans [3, 13]. Piperine on the other hand is present in black pepper and is a drug potentiator that inhibits human P-glycoprotein. Piperine was shown to have synergistic properties with rifampicin and decreased the number of bacteria in the lung of mice [12, 37, 38]. However, piperine is not currently used in the treatment of tuberculosis infected patients due to its likely and unpredictable drug-drug interactions with other co-administered medication. This study therefore aimed to identify alternatives for Rv1258c efflux pump inhibition.

The 3D structure of Rv1258c was predicted through homology modelling and validated using molecular dynamic simulation studies in a lipid bilayer consisting of POPE/POPG lipids. Pharmacophore modelling of Rv1258c-drug complexes was performed to search for new putative compounds and these were then validated by performing blind docking studies to Rv1258c. The Lowest Dope score protein model predicted for Rv1258c showed good consensus with our homologous template protein based on the structural comparison and a very low root mean square deviation between atoms suggesting that a correct fold was assigned to Rv1258c. Furthermore, the predicted model of Rv1258c passed all the quality evaluation tests suggesting that an reliable model was predicted. Additional validation of the model using molecular dynamics simulation indicated similar trajectory behaviours based on RMSD, RMSD per-residue and SASA analysis between Rv1258c and its homologous template 1pw4, providing support for template selection. The homologous template however showed lower RMSD, RMSD per-residue and SASA suggesting that it is less flexible and more compact than the predicted 3D model of Rv1258c. The PCA analysis affirmed previous statistics that Rv1258c explored a larger area of the phase space suggesting that Rv1258c is more flexible and dynamic in comparison to the template 1pw4 as was seen by analysis of the variance data. Interestingly, the first four PCA's contributed significantly to the mobility of Rv1258c. Another important finding of our study is the identification of one local energy minima state of the apo protein Rv1258c, which suggests that the structure of Rv1258c is highly flexible and dynamic. However, a limitation of this study is the use of only two principal components (Cartesian coordinates) to estimate the correct rugged free energy landscape that may result in artifacts due to the mixing of internal coordinates and overall motion of the protein [39]. Also, using only two-dimensional representations to estimate the free energy landscape of biomolecules may result in missing numerous metastable conformations of the protein because of minima overlapping [40]. The resolution here is to use dihedral angle PCA analysis using internal coordinates such as bond lengths and dihedral angles for dimensional reduction and including more than two PCA components to enhance minima detection. Another option is to perform replica exchange dynamics for longer simulation durations where the temperature is increased systematically to overcome high energy barriers. We selected one of the clusters which represented a potential metastable conformation for our docking studies. The result for the blind docking study of the five small molecules confirmed piperine and verapamil as the strongest binders to Rv1258c. They also bound to the same location within the binding cleft while spectinamide bound to a different location on the outside surface of the protein suggesting its ability to avoid the efflux channel. Contrary to the study of Sharma and colleagues, (2010), we identified a different active site (confirmed by metaPocket 2.0 analysis, S39 Fig) located deep within a binding cleft pocket which is in an open channel conformation. In our study no hydrogen bond interactions are found but rather non-polar contacts which are crucial for the binding of piperine and verapamil. This is foreseeable as we used a different homologous template (1pw4) and simulated the Rv1258c apo protein structure in a lipid bilayer of POPE/POPG. Subsequently, we leveraged one of the open states in docking studies and then used the high binding complexes in a pharmacophore search against the ZINC database, identifying 548 possible compounds. Additional docking validation was performed to the Rv1258c cluster and 246 compounds showed higher binding affinities than the known inhibitors piperine and verapamil. In contrast to piperine and verapamil the top 20 compounds made important hydrogen bonds (Ser26, Ser45 and Glu243), pi-pi stacking (Trp32) and a number of hydrophobic interactions that accounted for the higher affinity for Rv1258c. The top compounds will now be analysed and a number selected, purchased and tested *in vitro* to determine their ability to restore sensitivity of H37rV to rifampicin by inhibiting the efflux pump Rv1258c. These compounds could serve as alternatives to piperine and verapamil in the combination treatment of multi-drug resistant tuberculosis strains and could provide lead structures for further drug development.

## Conclusions

This study predicted the 3D-structure of the multi-drug efflux pump protein Rv1258c that is important to model protein-drug interactions. The 3D-structures of Rv1258c and the homologous template 1pw4 were also further utilized in molecular dynamic simulation studies within a POPE/POPG lipid bilayer and showed similar kinetic behaviour based on dynamics. Extraction of one cluster that represented a metastable conformation of Rv1258c allowed the identification of a putative binding site within a binding cleft when the protein channel has an open conformation. We identified 246 putative ligands using docking studies and also validated residues Ser26, Ser45, Glu243, Trp32 as crucial interaction partners responsible for the higher affinity. A selection of the top compounds will be purchased to validate them *in vitro* as potential modulators of antimycobacterial treatment. The implications of this study will provide alternatives to piperine and verapamil for Tuberculosis treatment to improve the bacterium’s susceptibility to various antimicrobials by inhibiting the efflux pump Rv1258c.

## Supporting information

S1 Fig Three dimensional structure of the LDSM predicted for Rv1258c. Histidine residues shown as sticks.
(TIFF)

S2 Fig Superimposition of the LDSM Rv1258c (cyan) on PDBID: 2GFP (green). (TIFF)

S3 Fig Superimposition of the homologous template 1pw4 (magenta) on PDBID: 2GFP (green). (TIFF)

S4 Fig DOPE score profile for the LDSM of Rv1258c (green) and the homologous template 1pw4 (red). (TIFF)

S5 Fig Clustering of the top 5 binding modes of piperine to cluster 7 of Rv1258c at an exhaustive value of 16. Piperine is shown as sticks and residues of Rv1258c are coloured by type using the python script “resicolor.py”. Colour schemes: acidic residues are red, basic in blue, nonpolar in orange, polar in green, cysteine residues in yellow and backbone atoms white.
(PNG)

S6 Fig Clustering of the top 5 binding modes of verapamil to cluster 7 of Rv1258c at an exhaustive value of 16. Verapamil is shown as sticks and residues of Rv1258c are coloured by type using the python script “resicolor.py”. Colour schemes: acidic residues are red, basic in blue, nonpolar in orange, polar in green, cysteine residues in yellow and backbone atoms white.
(PNG)

S7 Fig Clustering of the top 5 binding modes of piperine to cluster 7 of Rv1258c at an exhaustive value of 32. Piperine is shown as sticks and residues of Rv1258c are coloured by type using the python script “resicolor.py”. Colour schemes: acidic residues are red, basic in blue, nonpolar in orange, polar in green, cysteine residues in yellow and backbone atoms white.
(PNG)

S8 Fig Clustering of the top 5 binding modes of verapamil to cluster 7 of Rv1258c at an exhaustive value of 32. Verapamil is shown as sticks and residues of Rv1258c are coloured by type using the python script “resicolor.py”. Colour schemes: acidic residues are red, basic in blue, nonpolar in orange, polar in green, cysteine residues in yellow and backbone atoms white.
(PNG)

S9 Fig Clustering of the top 5 binding modes of piperine to cluster 7 of Rv1258c at an exhaustive value of 64. Piperine is shown as sticks and residues of Rv1258c are coloured by type using the python script “resicolor.py”. Colour schemes: acidic residues are red, basic in blue, nonpolar in orange, polar in green, cysteine residues in yellow and backbone atoms white.
(PNG)

S10 Fig Clustering of the top 5 binding modes of verapamil to cluster 7 of Rv1258c at an exhaustive value of 64. Verapamil is shown as sticks and residues of Rv1258c are coloured by type using the python script “resicolor.py”. Colour schemes: acidic residues are red, basic in blue, nonpolar in orange, polar in green, cysteine residues in yellow and backbone atoms white.
(PNG)

S11 Fig Clustering of the top 5 binding modes of piperine to cluster 7 of Rv1258c at an exhaustive value of 128. Piperine is shown as sticks and residues of Rv1258c are coloured by type using the python script “resicolor.py”. Colour schemes: acidic residues are red, basic in blue, nonpolar in orange, polar in green, cysteine residues in yellow and backbone atoms white.
(PNG)

S12 Fig Clustering of the top 5 binding modes of verapamil to cluster 7 of Rv1258c at an exhaustive value of 128. Verapamil is shown as sticks and residues of Rv1258c are coloured by type using the python script “resicolor.py”. Colour schemes: acidic residues are red, basic in blue, nonpolar in orange, polar in green, cysteine residues in yellow and backbone atoms white.
(PNG)

S13 Fig Pymol 3D interaction diagram showing piperine (red) bound to cluster 7 of Rv1258c. Hydrophobic interacting residues are labelled and coloured in magenta. Interactions calculated with binana.py python script (S1 Python Script).
(TIFF)

S14 Fig Pymol 3D interaction diagram showing verapamil (green) docked to cluster 7 of Rv1258c. Hydrophobic interacting residues are labelled and coloured in cyan/blue. Interactions calculated with binana.py python script (S1 Python Script).
(TIFF)

S15 Fig Poseview 2D interaction diagram showing chlopromazine docked to cluster 7 of Rv1258c. The dashed lines represent hydrogen bonds and the green spline segments illustrate hydrophobic contacts. (TIFF)

S16 Fig Poseview 2D interaction diagram showing spectinomycin docked to cluster 7 of Rv1258c. The dashed lines represent hydrogen bonds and the green spline segments illustrate hydrophobic contacts.
(TIFF)

S17 Fig Poseview 2D interaction diagram showing spectinamide_1599 docked to cluster 7 of Rv1258c. The dashed lines represent hydrogen bonds.
(TIFF)

S18 Fig Poseview 2D interaction diagram showing BNG docked to cluster 7 of Rv1258c. The dashed lines represent hydrogen bonds and the green spline segments illustrate hydrophobic contacts.
(TIFF)

S19 Fig Poseview 2D interaction diagram showing ZINC36652490 docked to cluster 7 of Rv1258c. The dashed lines represent hydrogen bonds and the green spline segments illustrate hydrophobic contacts and green dots represent pi-pi stacking interaction.
(PDF)

S20 Fig Poseview 2D interaction diagram showing ZINC16608089 docked to cluster 7 of Rv1258c. The dashed lines represent hydrogen bonds and the green spline segments illustrate hydrophobic contacts and green dots represent pi-pi stacking interaction.
(PDF)

S21 Fig Poseview 2D interaction diagram showing ZINC357294457 docked to cluster 7 of Rv1258c. The dashed lines represent hydrogen bonds and the green spline segments illustrate hydrophobic contacts and green dots represent pi-pi stacking interaction.
(PDF)

S22 Fig Poseview 2D interaction diagram showing ZINC35729461 docked to cluster 7 of Rv1258c. The dashed lines represent hydrogen bonds and the green spline segments illustrate hydrophobic contacts and green dots represent pi-pi stacking interaction.
(PDF)

S23 Fig Poseview 2D interaction diagram showing ZINC16608087 docked to cluster 7 of Rv1258c. The dashed lines represent hydrogen bonds and the green spline segments illustrate hydrophobic contacts and green dots represent pi-pi stacking interaction.
(PDF)

S24 Fig Poseview 2D interaction diagram showing ZINC15947760 docked to cluster 7 of Rv1258c. The dashed lines represent hydrogen bonds and the green spline segments illustrate hydrophobic contacts and green dots represent pi-pi stacking interaction.
(PDF)

S25 Fig Poseview 2D interaction diagram showing ZINC90733545 docked to cluster 7 of Rv1258c. The dashed lines represent hydrogen bonds and the green spline segments illustrate hydrophobic contacts and green dots represent pi-pi stacking interaction.
(PDF)

S26 Fig Poseview 2D interaction diagram showing ZINC45943747 docked to cluster 7 of Rv1258c. The dashed lines represent hydrogen bonds and the green spline segments illustrate hydrophobic contacts and green dots represent pi-pi stacking interaction.
(PDF)

S27 Fig Poseview 2D interaction diagram showing ZINC08920371 docked to cluster 7 of Rv1258c. The dashed lines represent hydrogen bonds and the green spline segments illustrate hydrophobic contacts and green dots represent pi-pi stacking interaction.
(PDF)

S28 Fig Poseview 2D interaction diagram showing ZINC72034391 docked to cluster 7 of Rv1258c. The dashed lines represent hydrogen bonds and the green spline segments illustrate hydrophobic contacts and green dots represent pi-pi stacking interaction.
(PDF)

S29 Fig Poseview 2D interaction diagram showing ZINC45943751 docked to cluster 7 of Rv1258c. The dashed lines represent hydrogen bonds and the green spline segments illustrate hydrophobic contacts and green dots represent pi-pi stacking interaction.
(PDF)

S30 Fig Poseview 2D interaction diagram showing ZINC16608126 docked to cluster 7 of Rv1258c. The dashed lines represent hydrogen bonds and the green spline segments illustrate hydrophobic contacts and green dots represent pi-pi stacking interaction.
(PDF)

S31 Fig Poseview 2D interaction diagram showing ZINC08920366 docked to cluster 7 of Rv1258c. The dashed lines represent hydrogen bonds and the green spline segments illustrate hydrophobic contacts and green dots represent pi-pi stacking interaction.
(PDF)

S32 Fig Poseview 2D interaction diagram showing ZINC06793104 docked to cluster 7 of Rv1258c. The dashed lines represent hydrogen bonds and the green spline segments illustrate hydrophobic contacts and green dots represent pi-pi stacking interaction.
(PDF)

S33 Fig Poseview 2D interaction diagram showing ZINC45943745 docked to cluster 7 of Rv1258c. The dashed lines represent hydrogen bonds and the green spline segments illustrate hydrophobic contacts and green dots represent pi-pi stacking interaction.
(PDF)

S34 Fig Poseview 2D interaction diagram showing ZINC09701714 docked to cluster 7 of Rv1258c. The dashed lines represent hydrogen bonds and the green spline segments illustrate hydrophobic contacts and green dots represent pi-pi stacking interaction.
(PDF)

S35 Fig Poseview 2D interaction diagram showing ZINC35933009 docked to cluster 7 of Rv1258c. The dashed lines represent hydrogen bonds and the green spline segments illustrate hydrophobic contacts and green dots represent pi-pi stacking interaction.
(PDF)

S36 Fig Poseview 2D interaction diagram showing ZINC78721136 docked to cluster 7 of Rv1258c. The dashed lines represent hydrogen bonds and the green spline segments illustrate hydrophobic contacts and green dots represent pi-pi stacking interaction.
(PDF)

S37 Fig Poseview 2D interaction diagram showing ZINC15947762 docked to cluster 7 of Rv1258c. The dashed lines represent hydrogen bonds and the green spline segments illustrate hydrophobic contacts and green dots represent pi-pi stacking interaction.
(PDF)

S38 Fig Poseview 2D interaction diagram showing ZINC16608150 docked to cluster 7 of Rv1258c. The dashed lines represent hydrogen bonds and the green spline segments illustrate hydrophobic contacts and green dots represent pi-pi stacking interaction.
(PDF)

S39 Fig Metapocket 2.0 predicted ligand binding sites for Rv1258c. The different coloured spheres represent the different methods employed by Metapocket: LIGSITE^CS^ (purple), PASS (cyan), SURFNET (brown), Q-SiteFinder (blue), Fpocket (pink), ConCavity (orange), GHECOM (yellow) and POCASA (wheat) are all from their top 1 prediction where ligands piperine (red) and verapamil (blue) bind. The MetaPocket site for Rv1258c is shown as a red sphere.
(PNG)

S40 Fig Lowest DOPE score model for Rv1258c predicted using MODELLER in protein databank format. (PDB)

S1 Text Configuration file for the docking parameters. (TXT)

S1 Python Script Python script to calculate protein drug interactions. (PY)

S1 Movie Simulation movie for 1pw4. Surface representation of 1pw4. https://drive.google.com/open?id=1YJuVMEH3xOVFBmCc_wzhlHrr-ul37KXJ.
(MPG)

S2 Movie Simulation movie for Rv1258c. Surface representation of Rv1258c. https://drive.google.com/open?id=1Zpy4GV6WIIVnmTw0oXWUiSSHEUtyJR32.
(MPG)
