# Correction: Molecular modelling and simulation studies of the *Mycobacterium tuberculosis* multidrug efflux pump protein Rv1258c

**DOI:** 10.1371/journal.pone.0209717

**Published:** 2018-12-19

**Authors:** The PLOS ONE Staff

## Notice of Republication

This article was republished on December 07, 2018 to correct errors to the ORCID iDs of the fourth and fifth authors introduced during the typesetting process. The publisher apologizes for the errors. Please download this article again to view the correct version.
