# Supplementary figures and images for: Molecular modelling and simulation studies of the Mycobacterium tuberculosis multidrug efflux pump protein Rv1258c
Source: PLoS One. 2018 Nov 26;13(11):e0207605. doi: 10.1371/journal.pone.0207605 (PMC6261026; doi:10.1371/journal.pone.0207605)

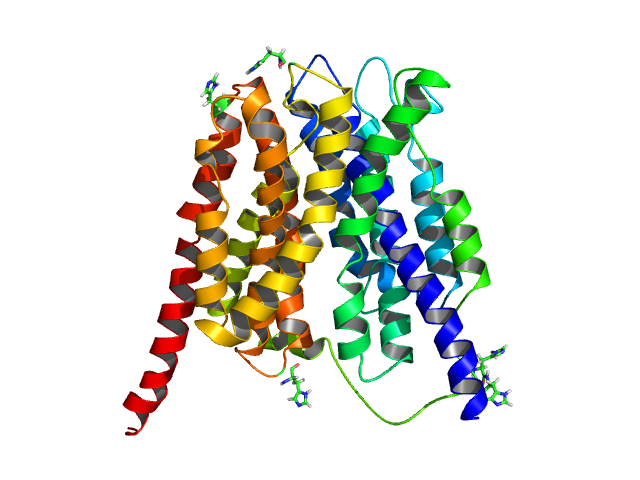

Supplement: S1 Fig — Histidine residues shown as sticks. (TIFF) [file pone.0207605.s001.tiff]

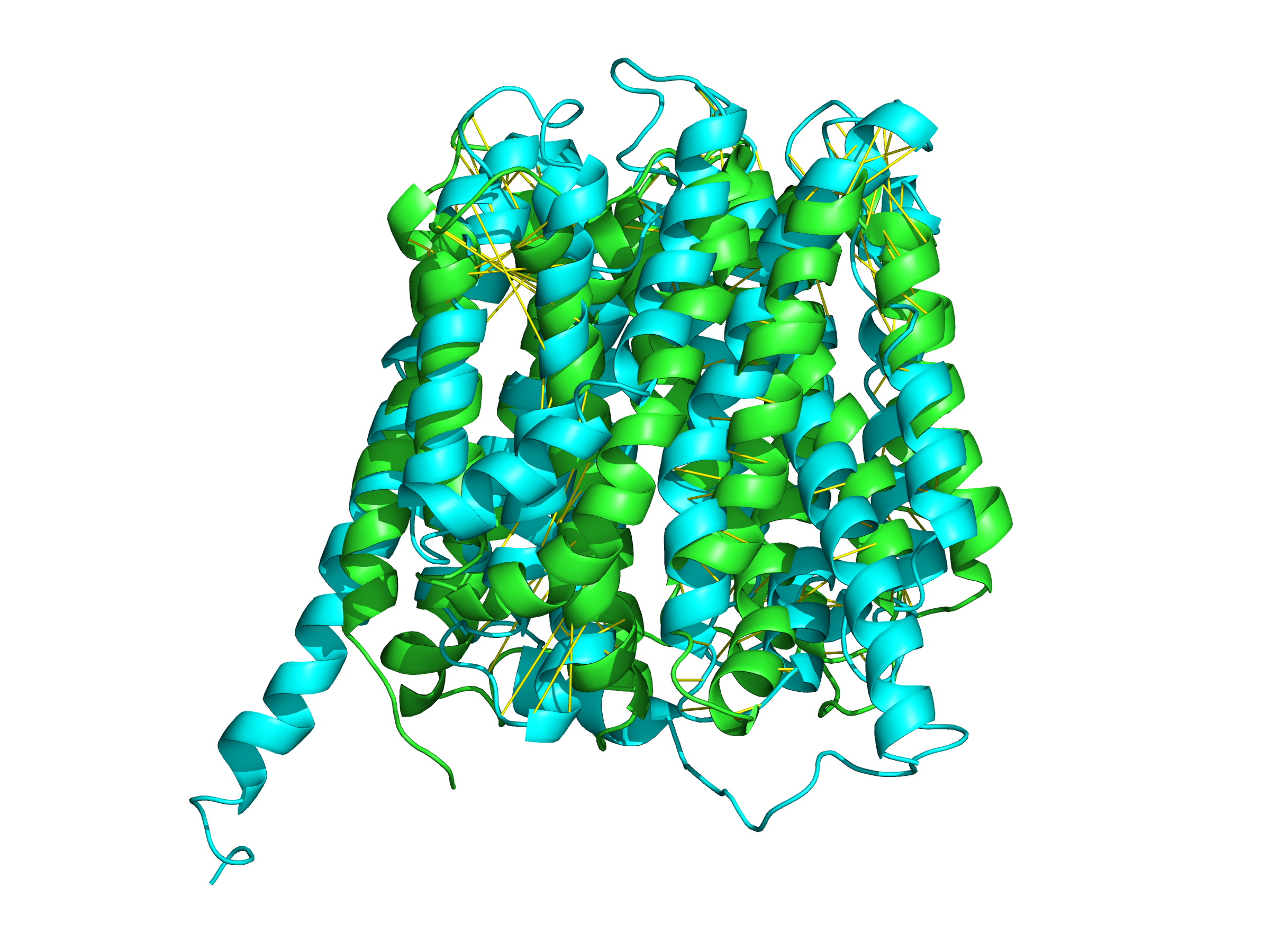

Supplement: S2 Fig — (TIFF) [file pone.0207605.s002.tiff]

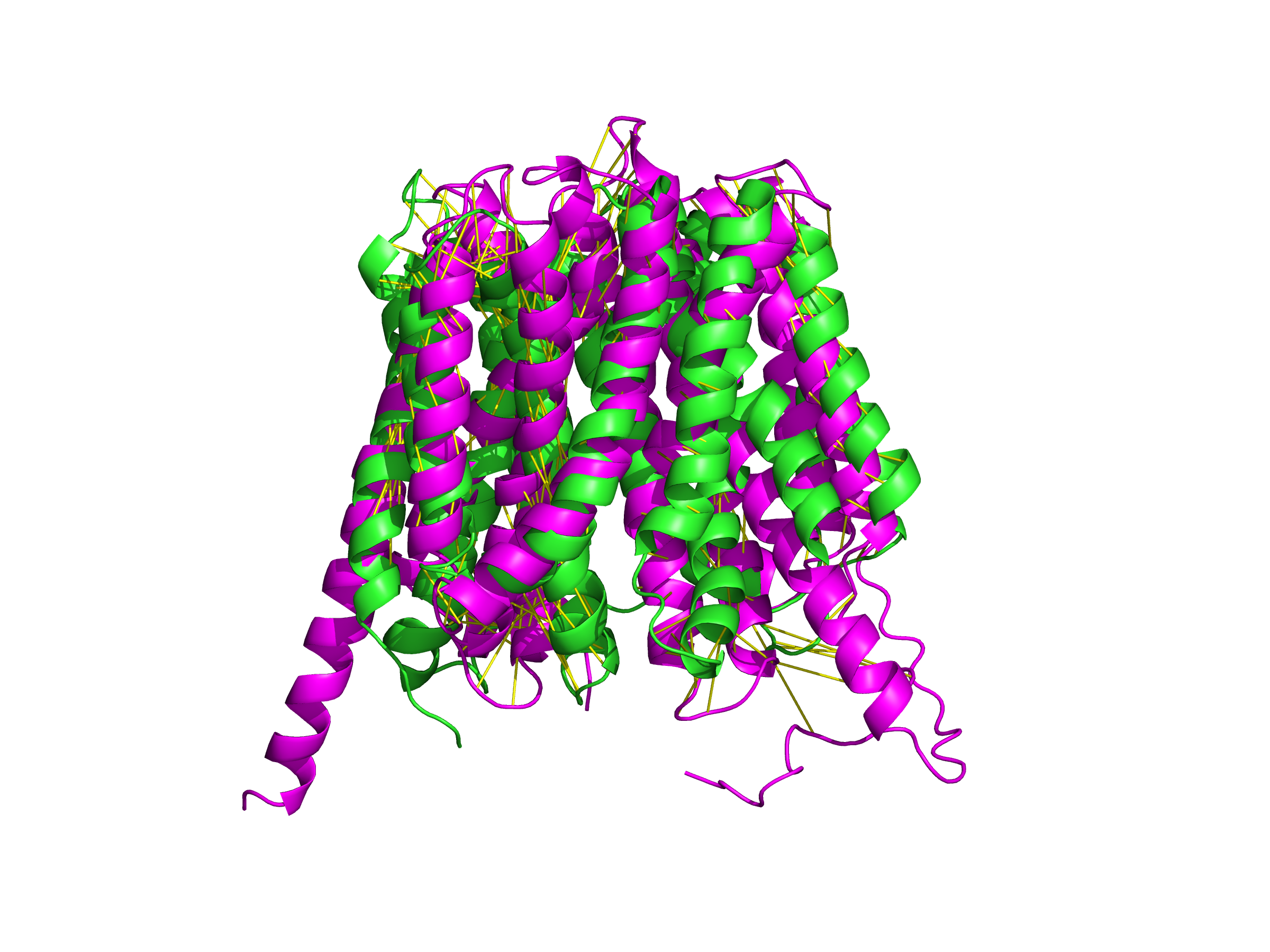

Supplement: S3 Fig — (TIFF) [file pone.0207605.s003.tiff]

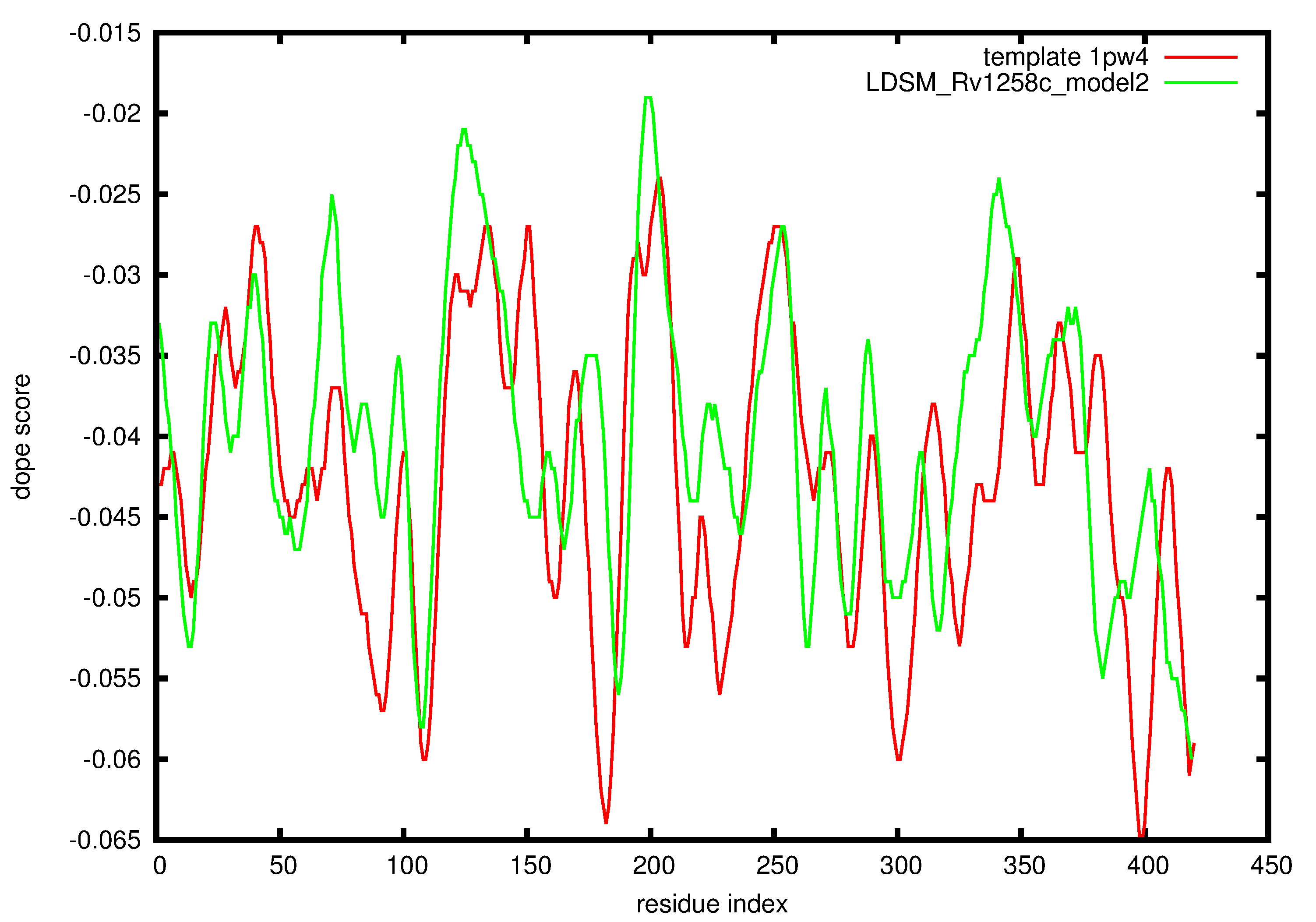

Supplement: S4 Fig — (TIFF) [file pone.0207605.s004.tiff]

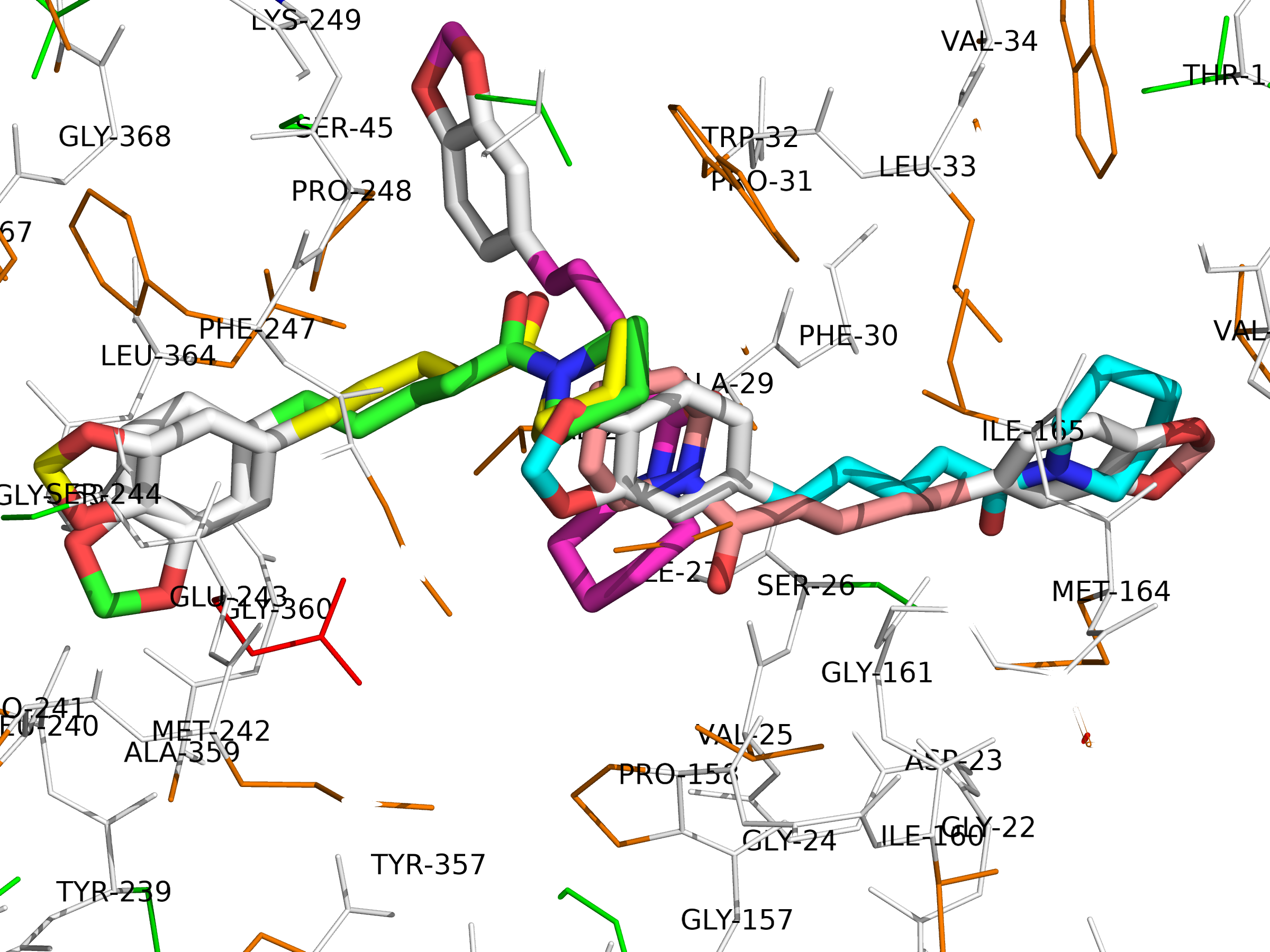

Supplement: S5 Fig — Piperine is shown as sticks and residues of Rv1258c are coloured by type using the python script “resicolor.py”. Colour schemes: acidic residues are red, basic in blue, nonpolar in orange, polar in green, cysteine residues in yellow and backbone atoms white. (PNG) [file pone.0207605.s005.png]

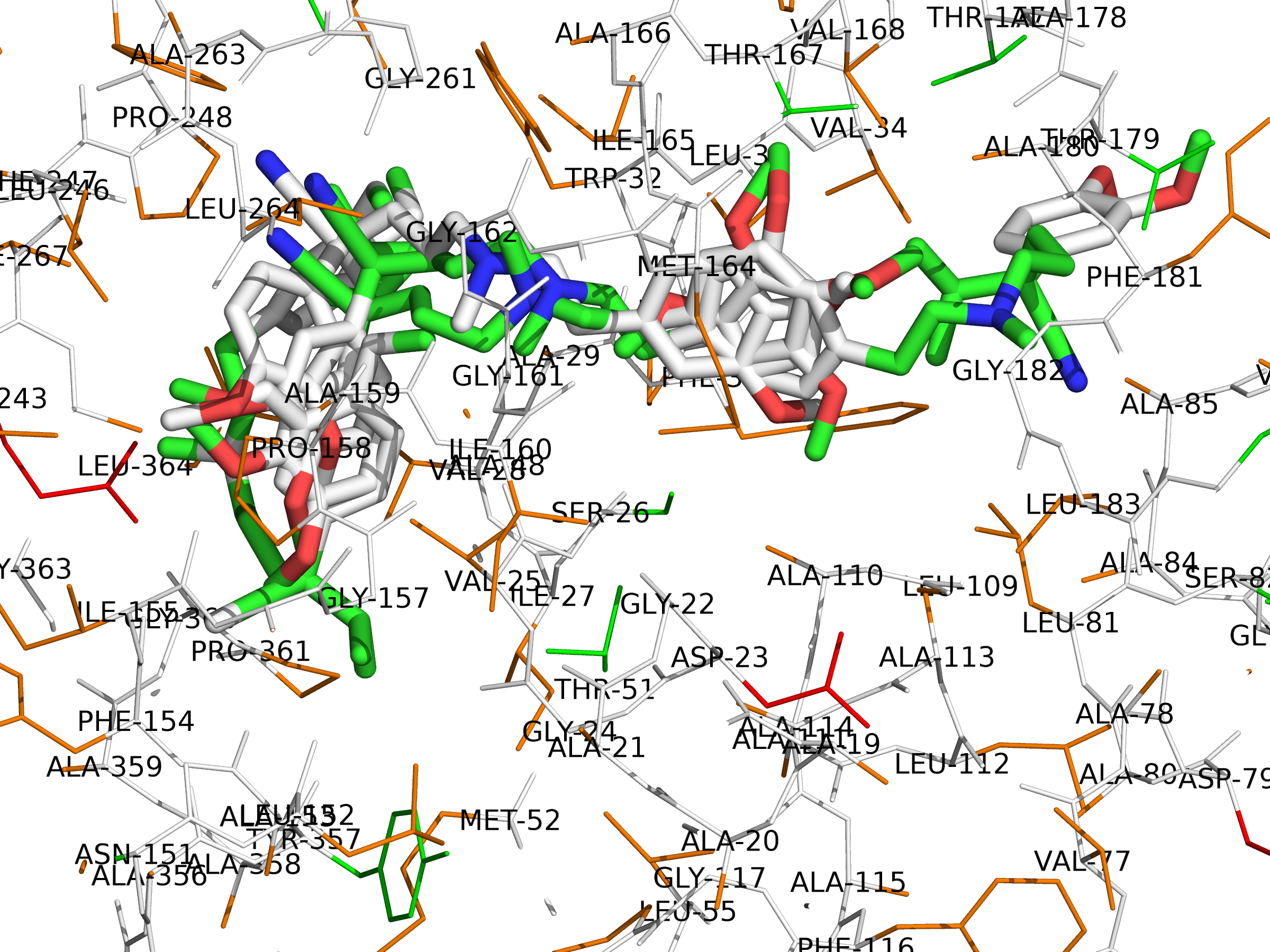

Supplement: S6 Fig — Verapamil is shown as sticks and residues of Rv1258c are coloured by type using the python script “resicolor.py”. Colour schemes: acidic residues are red, basic in blue, nonpolar in orange, polar in green, cysteine residues in yellow and backbone atoms white. (PNG) [file pone.0207605.s006.png]

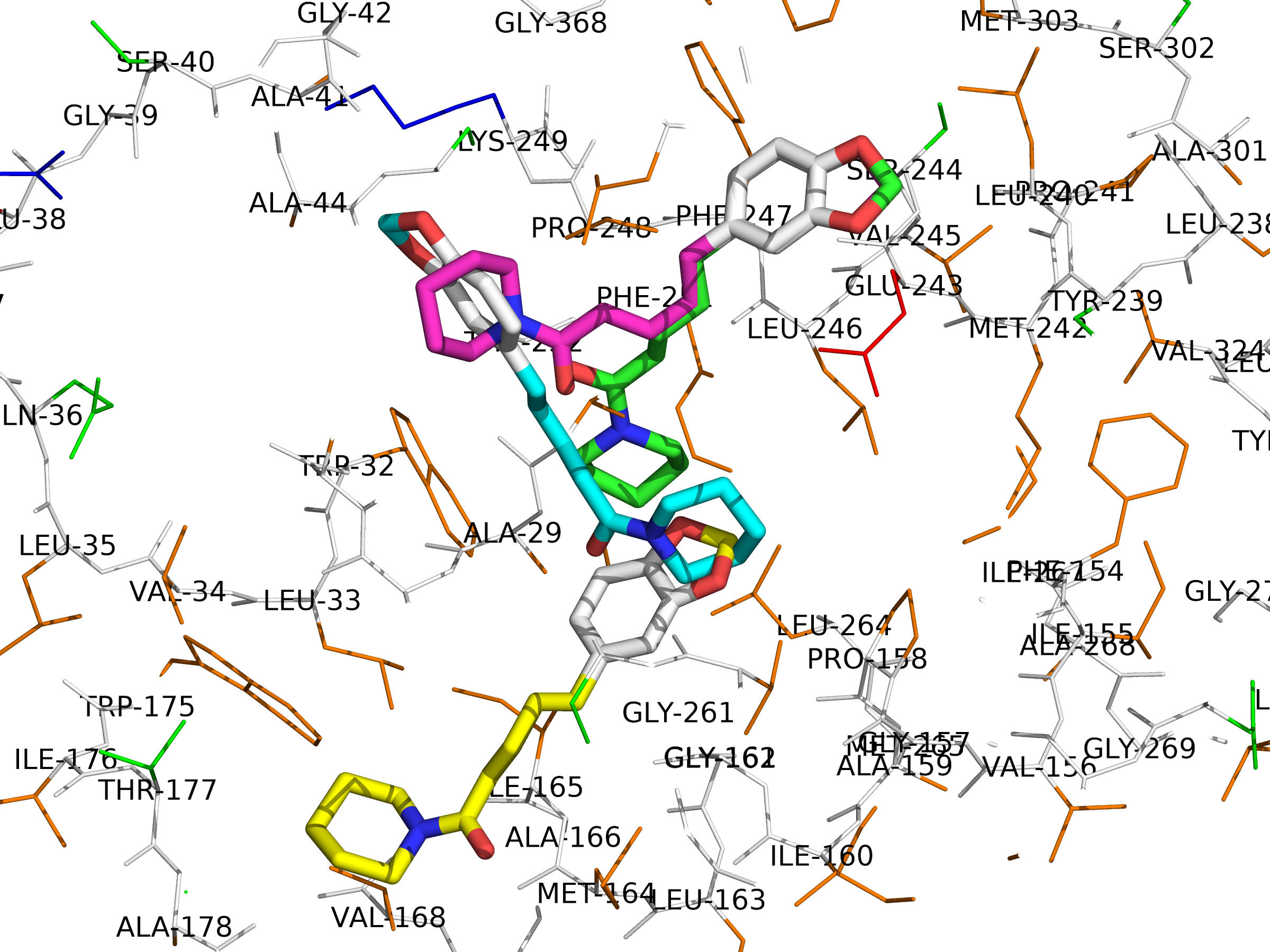

Supplement: S7 Fig — Piperine is shown as sticks and residues of Rv1258c are coloured by type using the python script “resicolor.py”. Colour schemes: acidic residues are red, basic in blue, nonpolar in orange, polar in green, cysteine residues in yellow and backbone atoms white. (PNG) [file pone.0207605.s007.png]

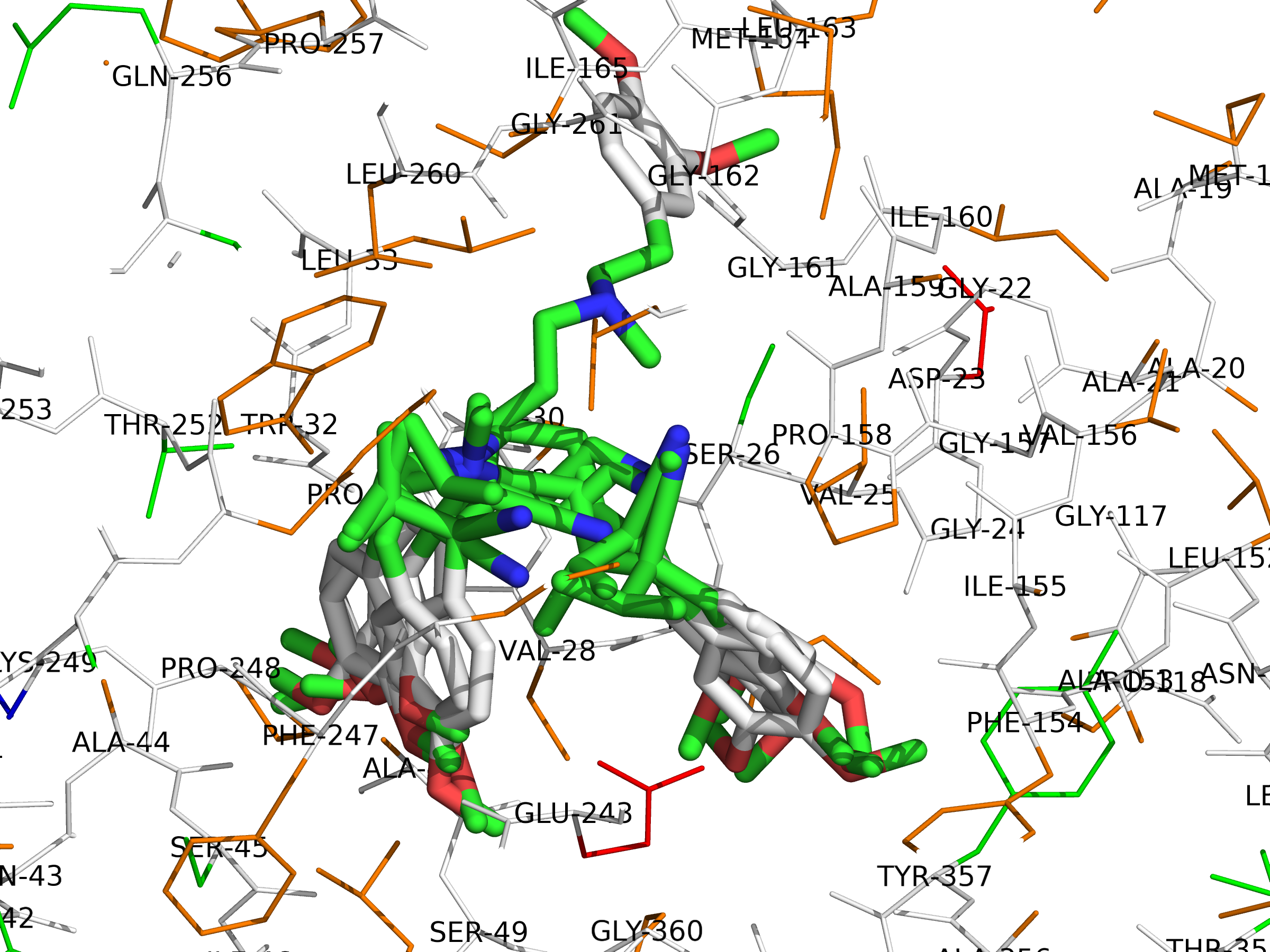

Supplement: S8 Fig — Verapamil is shown as sticks and residues of Rv1258c are coloured by type using the python script “resicolor.py”. Colour schemes: acidic residues are red, basic in blue, nonpolar in orange, polar in green, cysteine residues in yellow and backbone atoms white. (PNG) [file pone.0207605.s008.png]

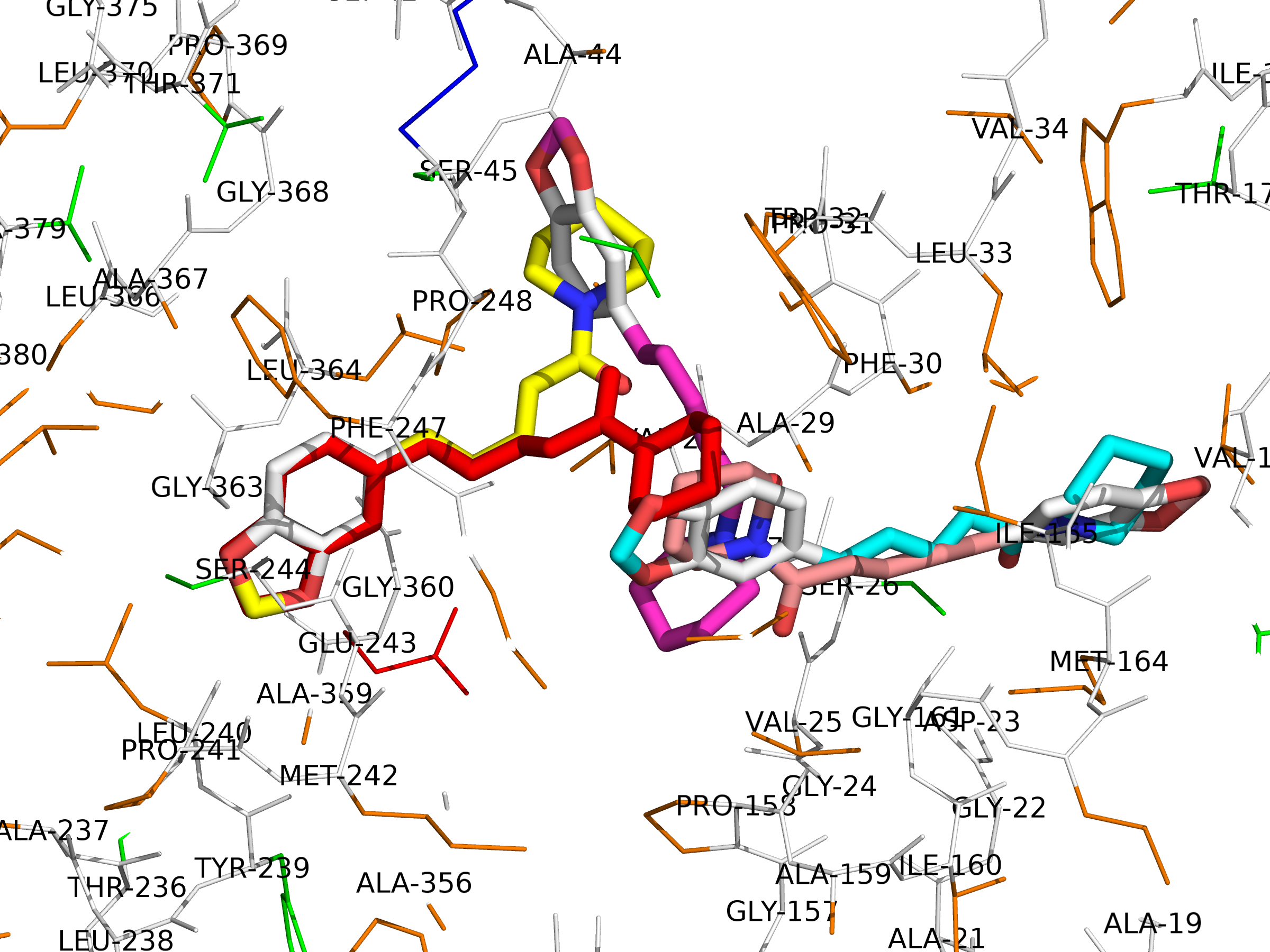

Supplement: S9 Fig — Piperine is shown as sticks and residues of Rv1258c are coloured by type using the python script “resicolor.py”. Colour schemes: acidic residues are red, basic in blue, nonpolar in orange, polar in green, cysteine residues in yellow and backbone atoms white. (PNG) [file pone.0207605.s009.png]

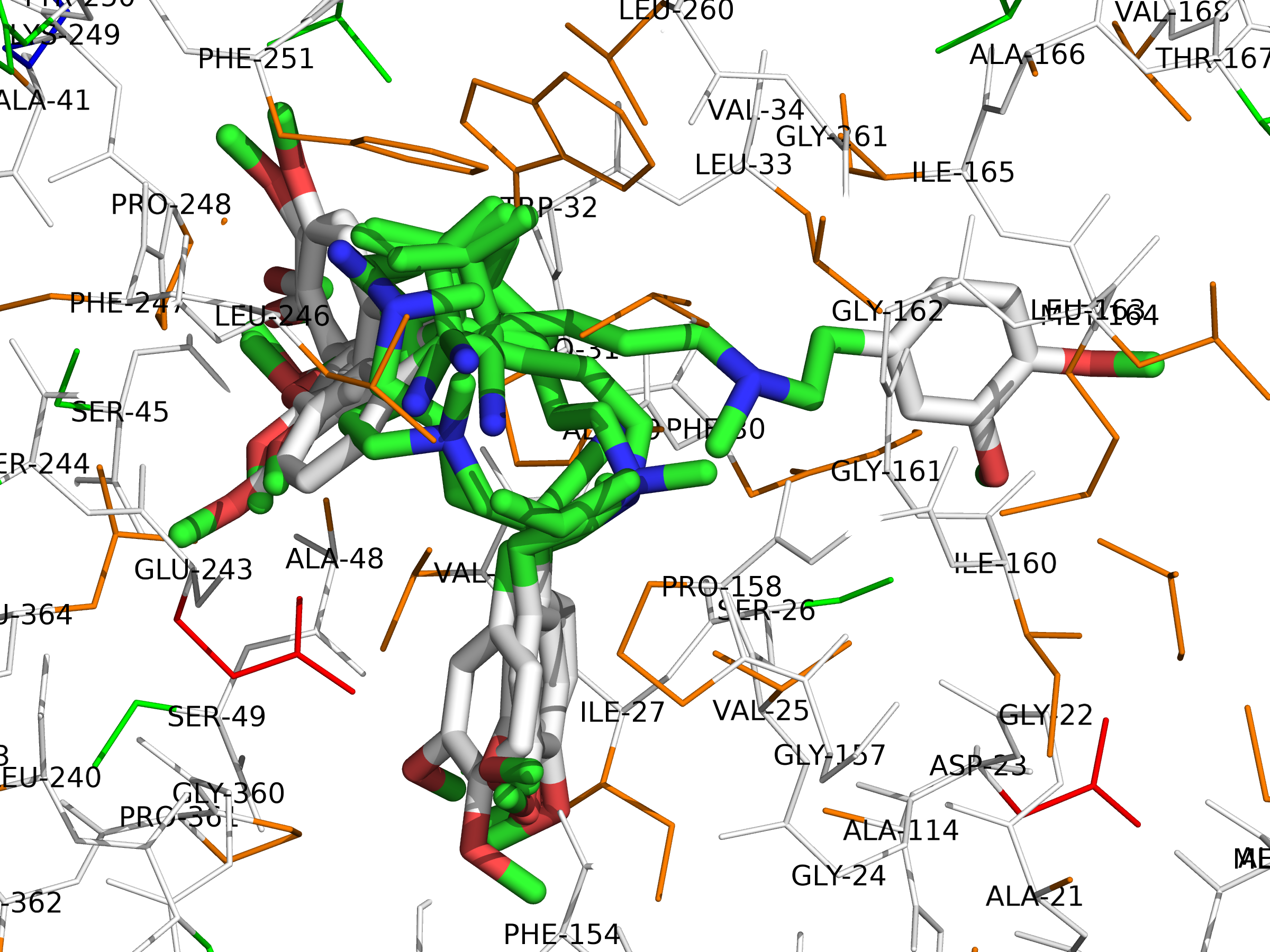

Supplement: S10 Fig — Verapamil is shown as sticks and residues of Rv1258c are coloured by type using the python script “resicolor.py”. Colour schemes: acidic residues are red, basic in blue, nonpolar in orange, polar in green, cysteine residues in yellow and backbone atoms white. (PNG) [file pone.0207605.s010.png]

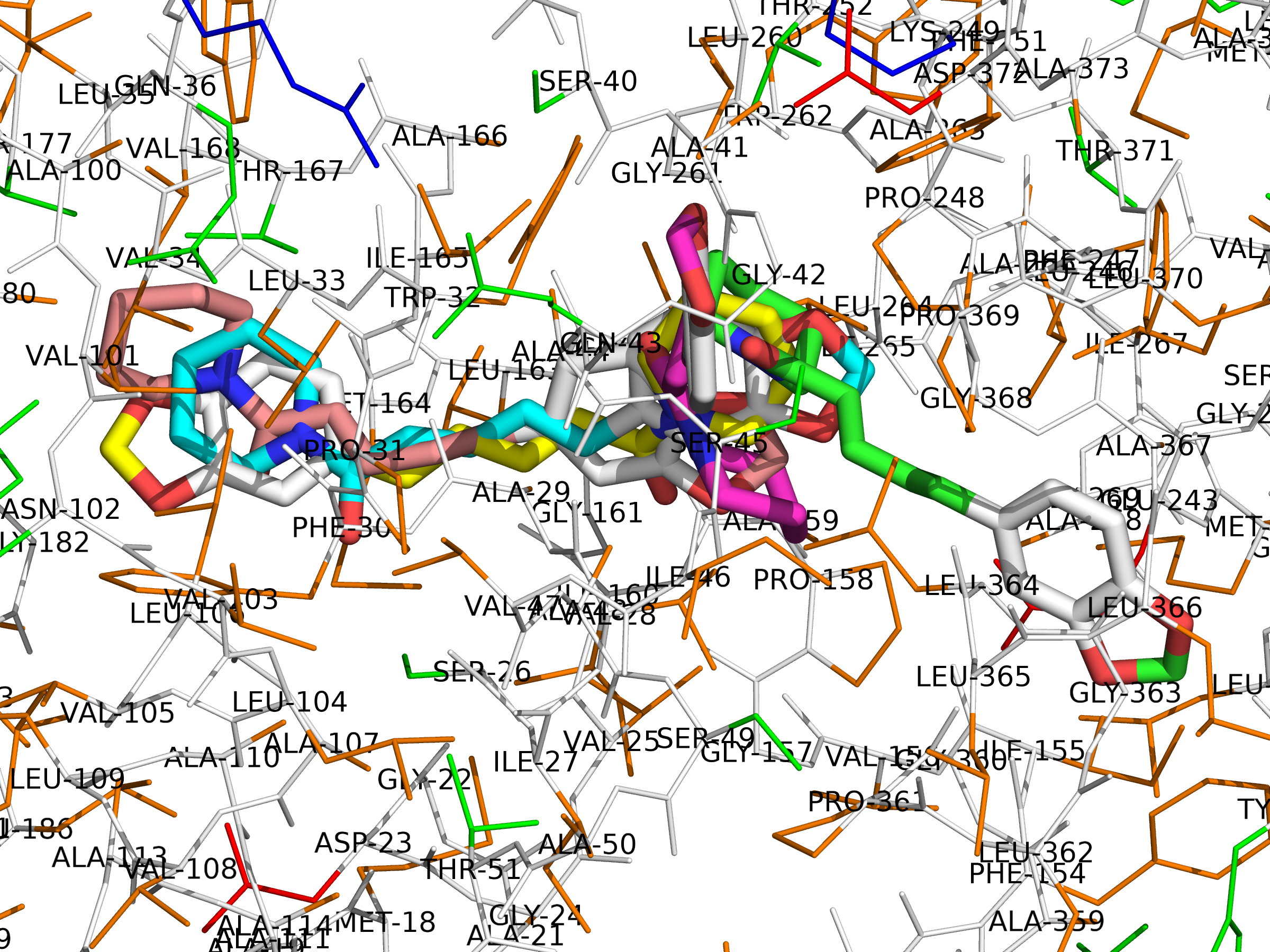

Supplement: S11 Fig — Piperine is shown as sticks and residues of Rv1258c are coloured by type using the python script “resicolor.py”. Colour schemes: acidic residues are red, basic in blue, nonpolar in orange, polar in green, cysteine residues in yellow and backbone atoms white. (PNG) [file pone.0207605.s011.png]

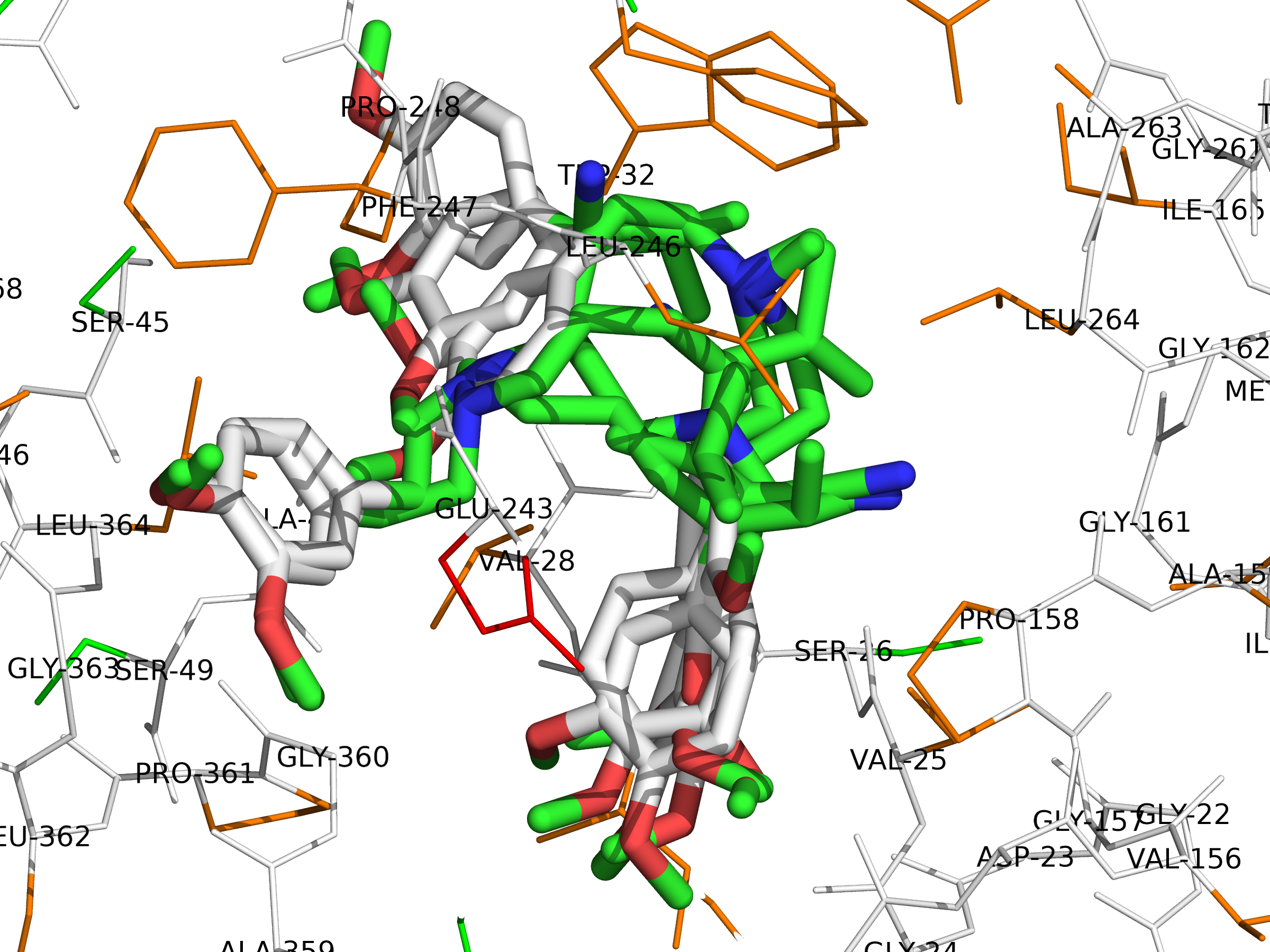

Supplement: S12 Fig — Verapamil is shown as sticks and residues of Rv1258c are coloured by type using the python script “resicolor.py”. Colour schemes: acidic residues are red, basic in blue, nonpolar in orange, polar in green, cysteine residues in yellow and backbone atoms white. (PNG) [file pone.0207605.s012.png]

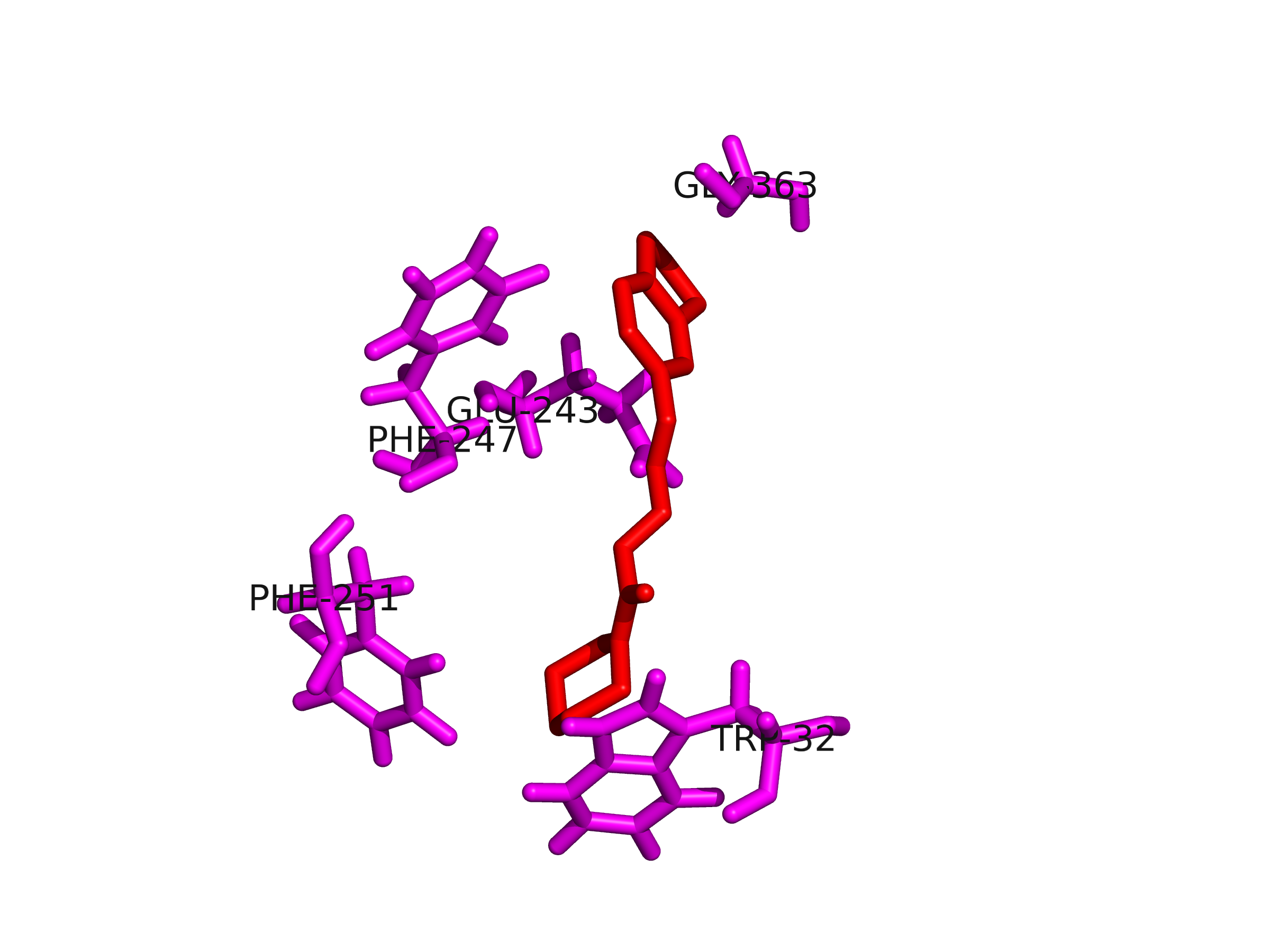

Supplement: S13 Fig — Hydrophobic interacting residues are labelled and coloured in magenta. Interactions calculated with binana.py python script (S1 Python Script). (TIFF) [file pone.0207605.s013.tiff]

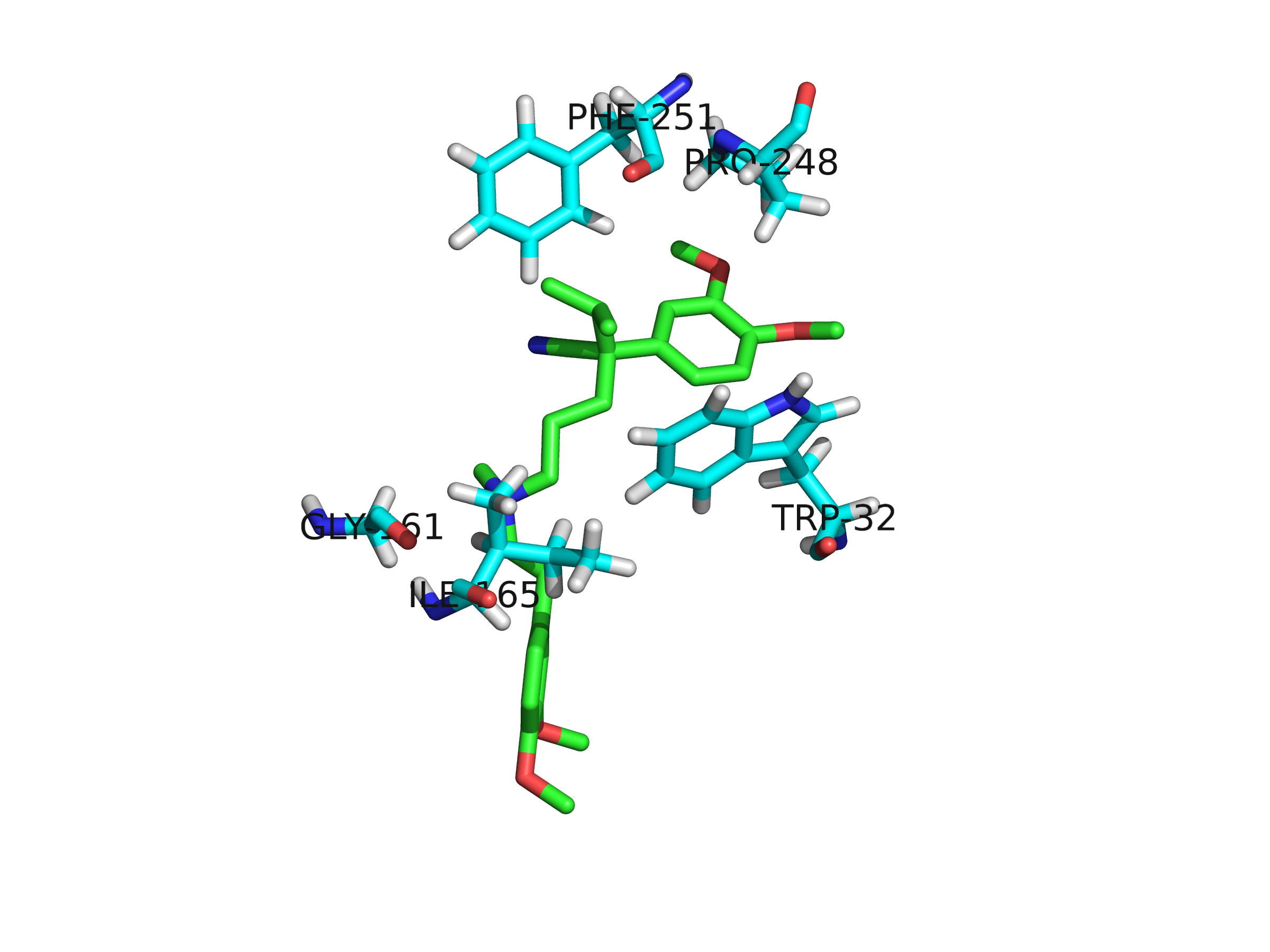

Supplement: S14 Fig — Hydrophobic interacting residues are labelled and coloured in cyan/blue. Interactions calculated with binana.py python script (S1 Python Script). (TIFF) [file pone.0207605.s014.tiff]

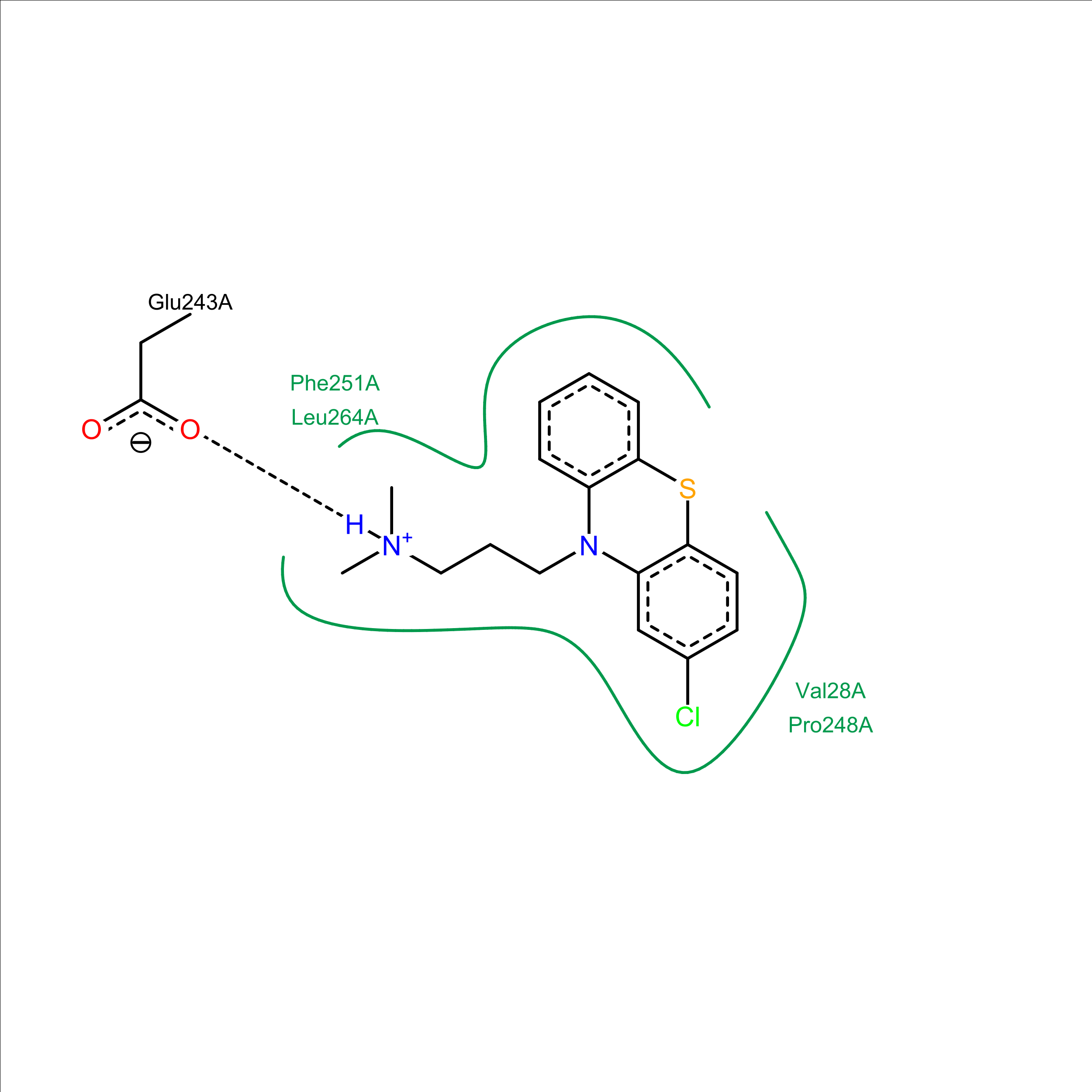

Supplement: S15 Fig — (TIFF) [file pone.0207605.s015.tiff]

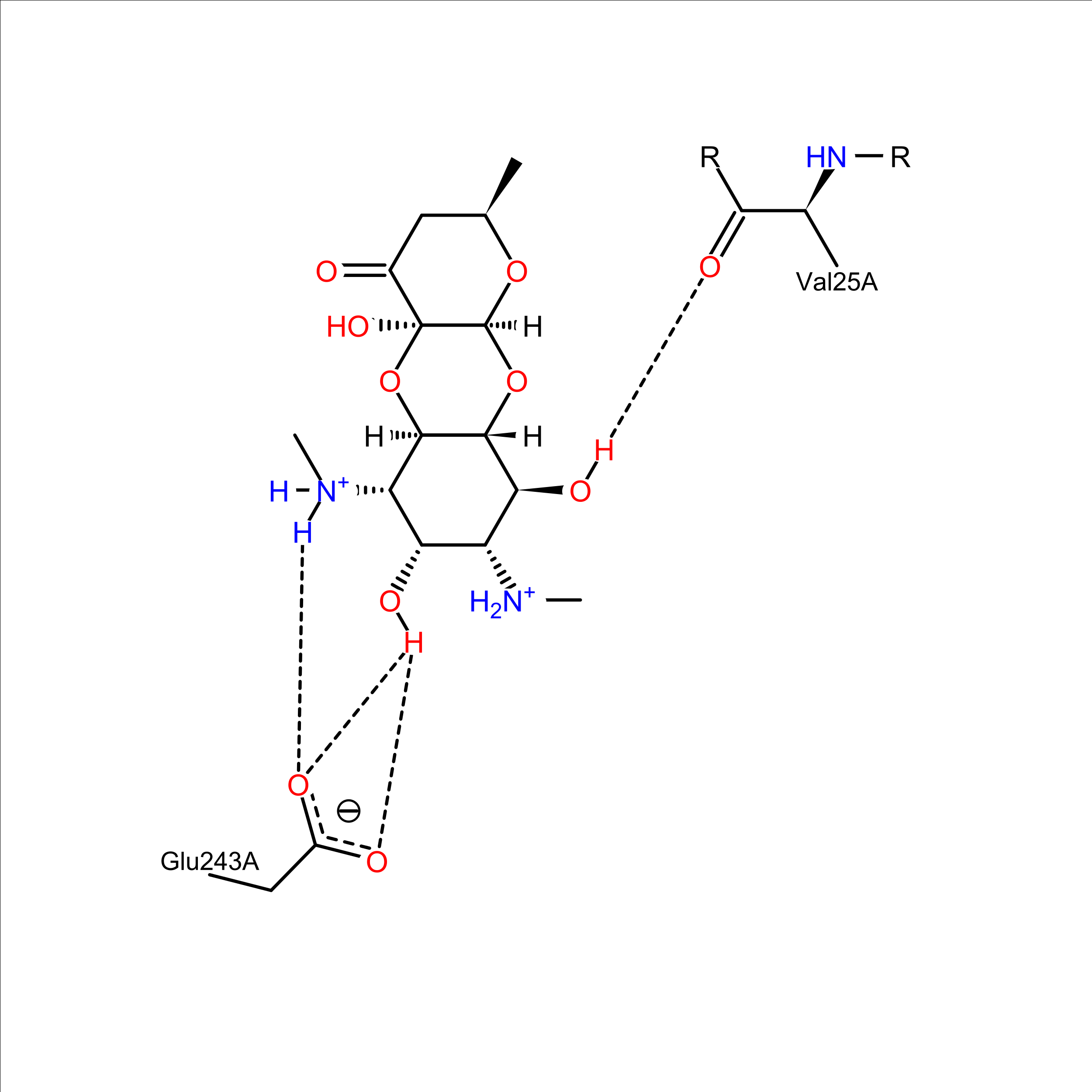

Supplement: S16 Fig — The dashed lines represent hydrogen bonds and the green spline segments illustrate hydrophobic contacts. (TIFF) [file pone.0207605.s016.tiff]

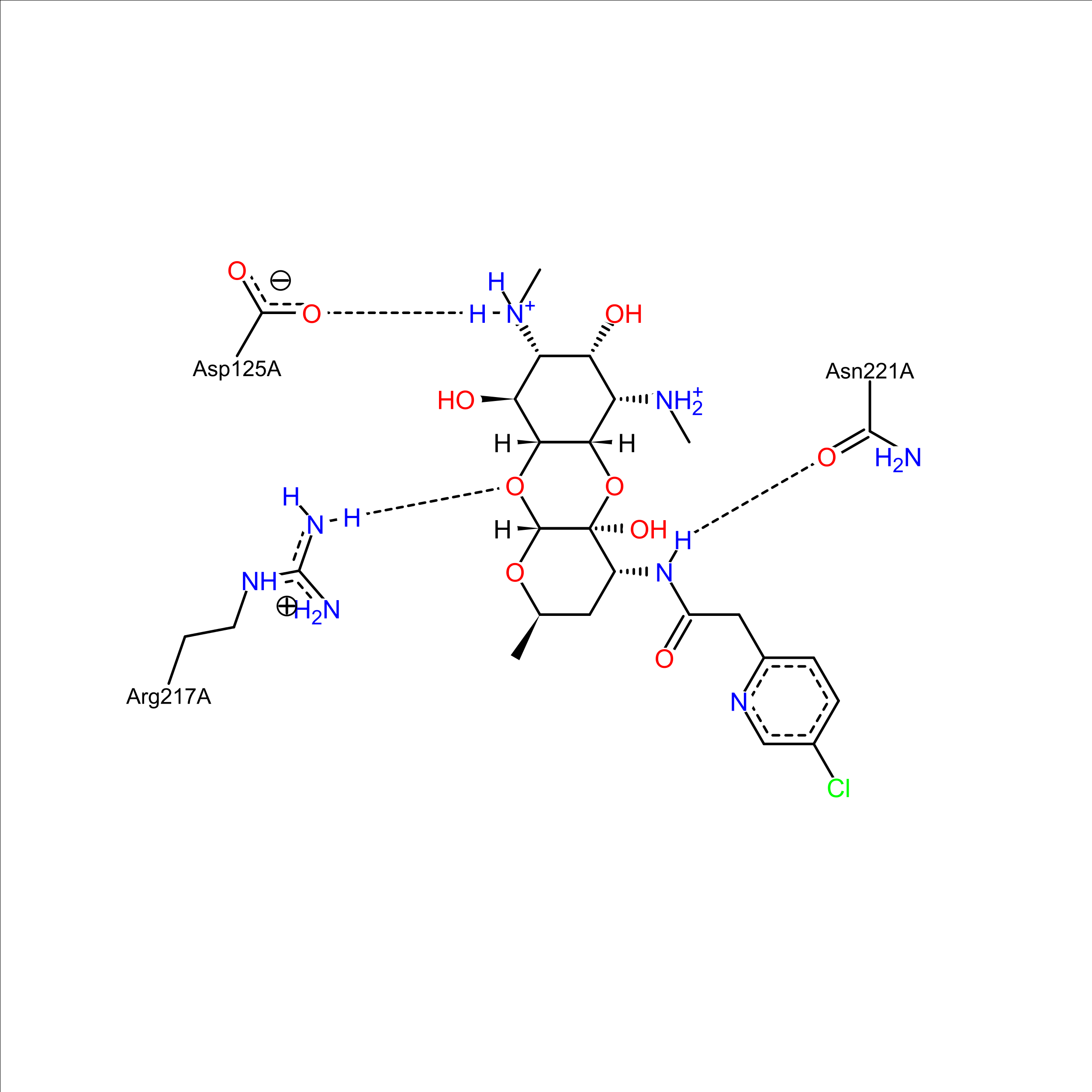

Supplement: S17 Fig — The dashed lines represent hydrogen bonds. (TIFF) [file pone.0207605.s017.tiff]

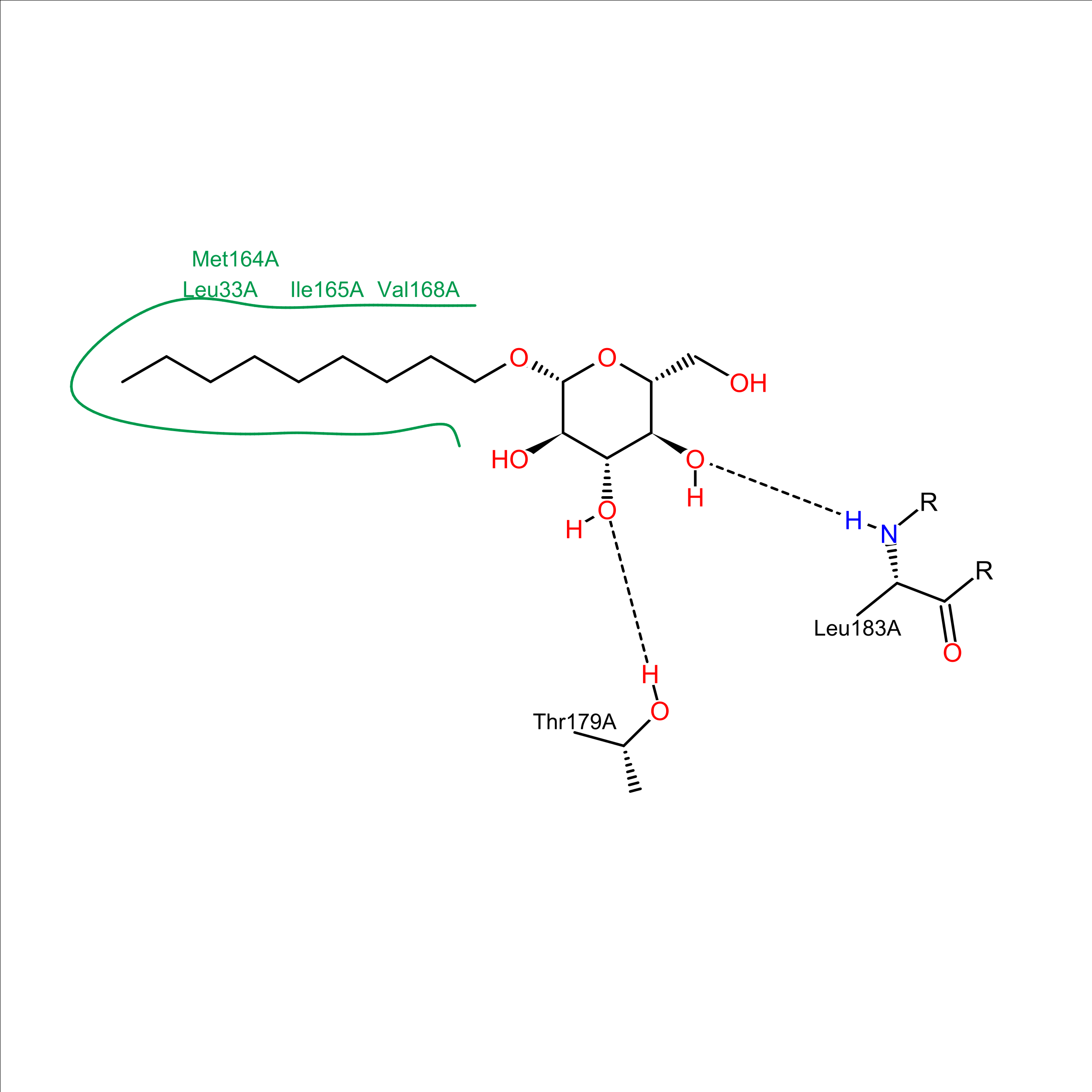

Supplement: S18 Fig — The dashed lines represent hydrogen bonds and the green spline segments illustrate hydrophobic contacts. (TIFF) [file pone.0207605.s018.tiff]

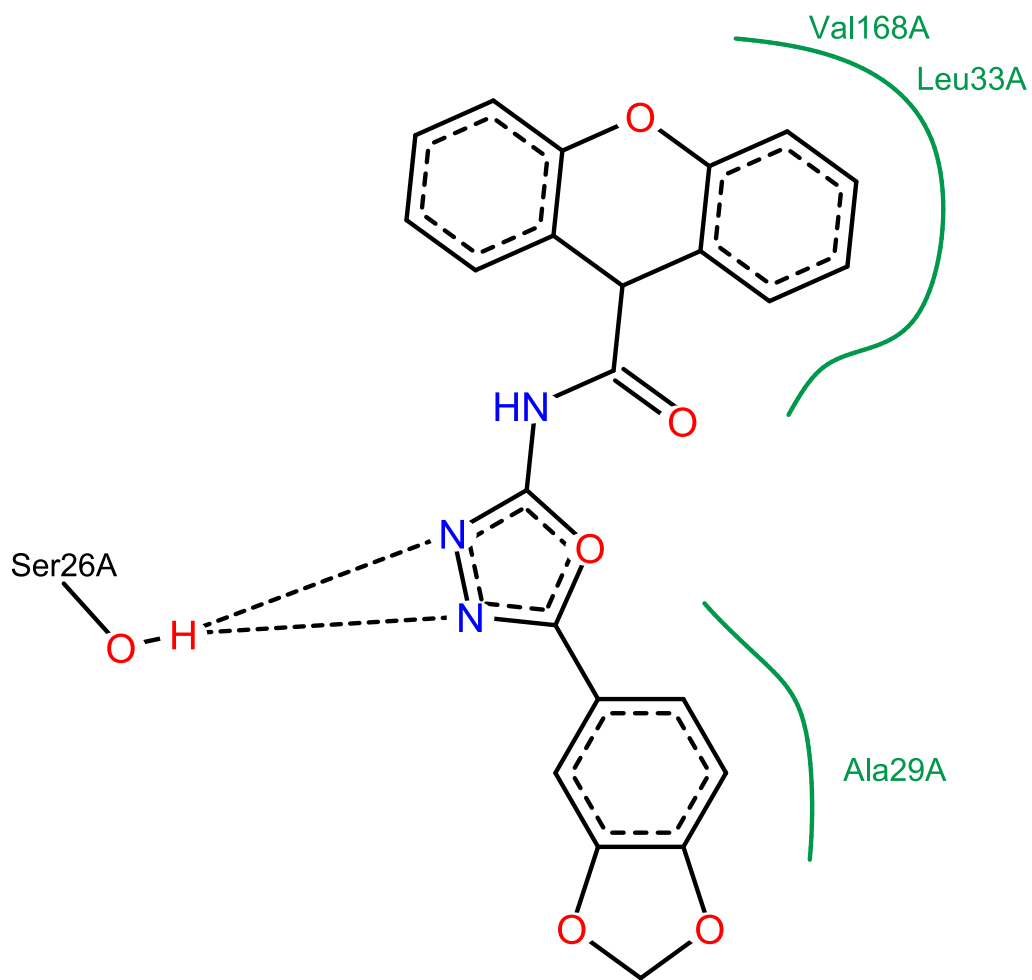

Supplement: S19 Fig — The dashed lines represent hydrogen bonds and the green spline segments illustrate hydrophobic contacts and green dots represent pi-pi stacking interaction. (PDF) [file pone.0207605.s019.pdf]

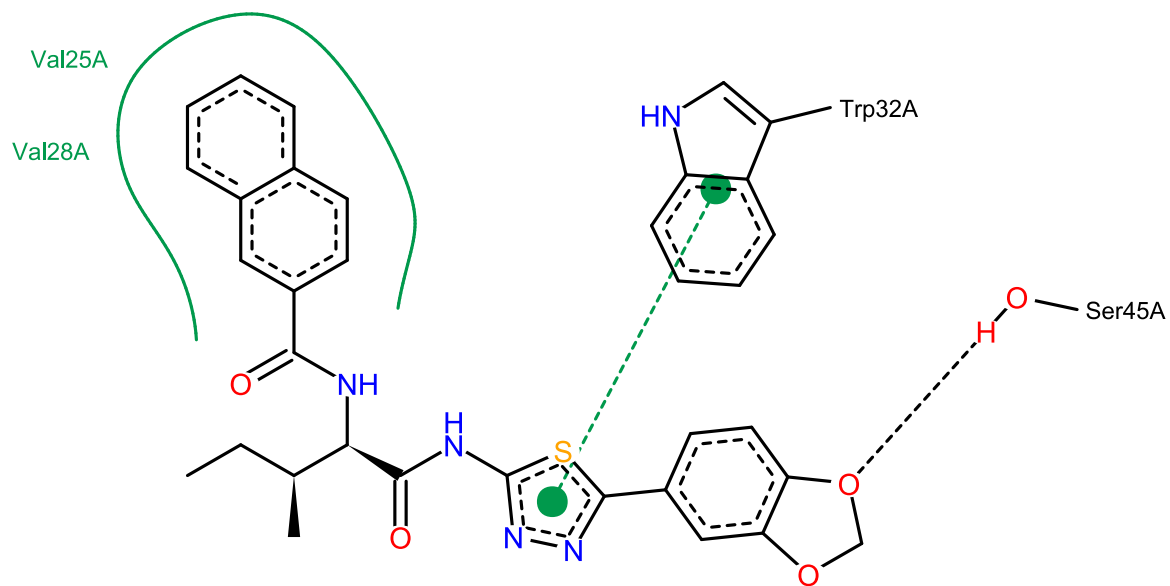

Supplement: S20 Fig — The dashed lines represent hydrogen bonds and the green spline segments illustrate hydrophobic contacts and green dots represent pi-pi stacking interaction. (PDF) [file pone.0207605.s020.pdf]

Ser45A

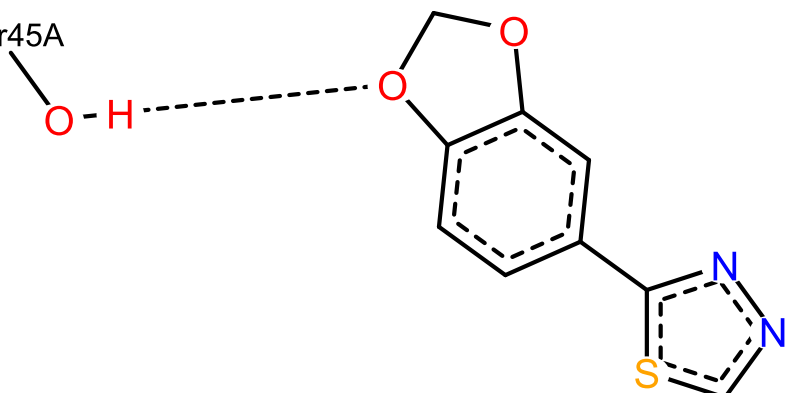

Glu243A

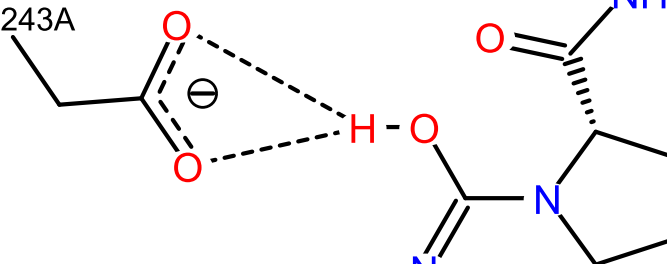

Pro158A

Val25A

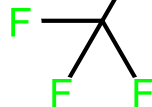

Supplement: S21 Fig — The dashed lines represent hydrogen bonds and the green spline segments illustrate hydrophobic contacts and green dots represent pi-pi stacking interaction. (PDF) [file pone.0207605.s021.pdf]

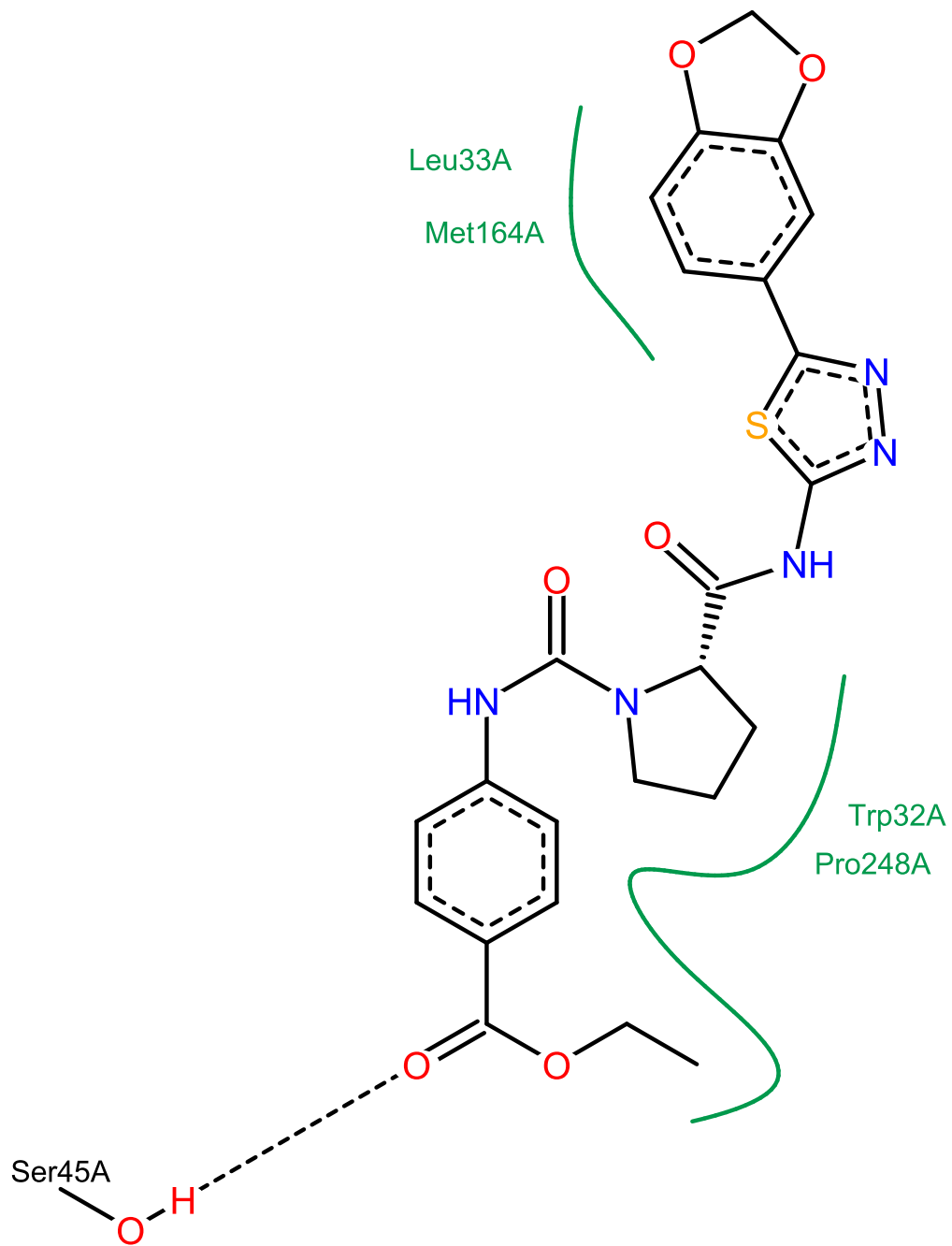

Supplement: S22 Fig — The dashed lines represent hydrogen bonds and the green spline segments illustrate hydrophobic contacts and green dots represent pi-pi stacking interaction. (PDF) [file pone.0207605.s022.pdf]

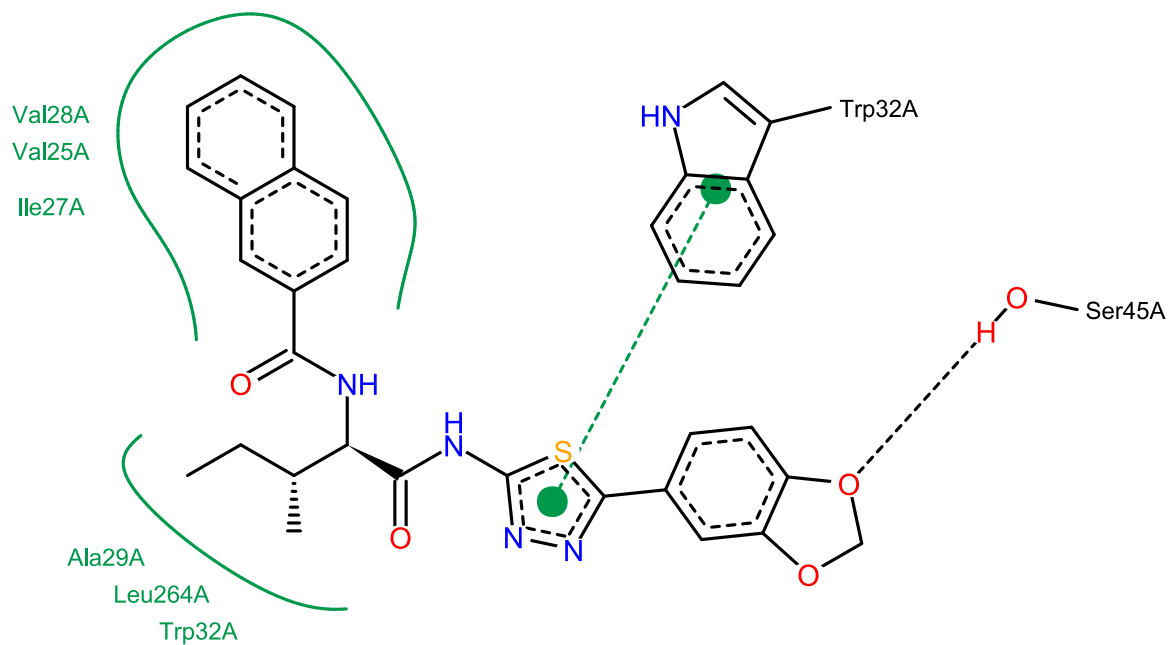

Supplement: S23 Fig — The dashed lines represent hydrogen bonds and the green spline segments illustrate hydrophobic contacts and green dots represent pi-pi stacking interaction. (PDF) [file pone.0207605.s023.pdf]

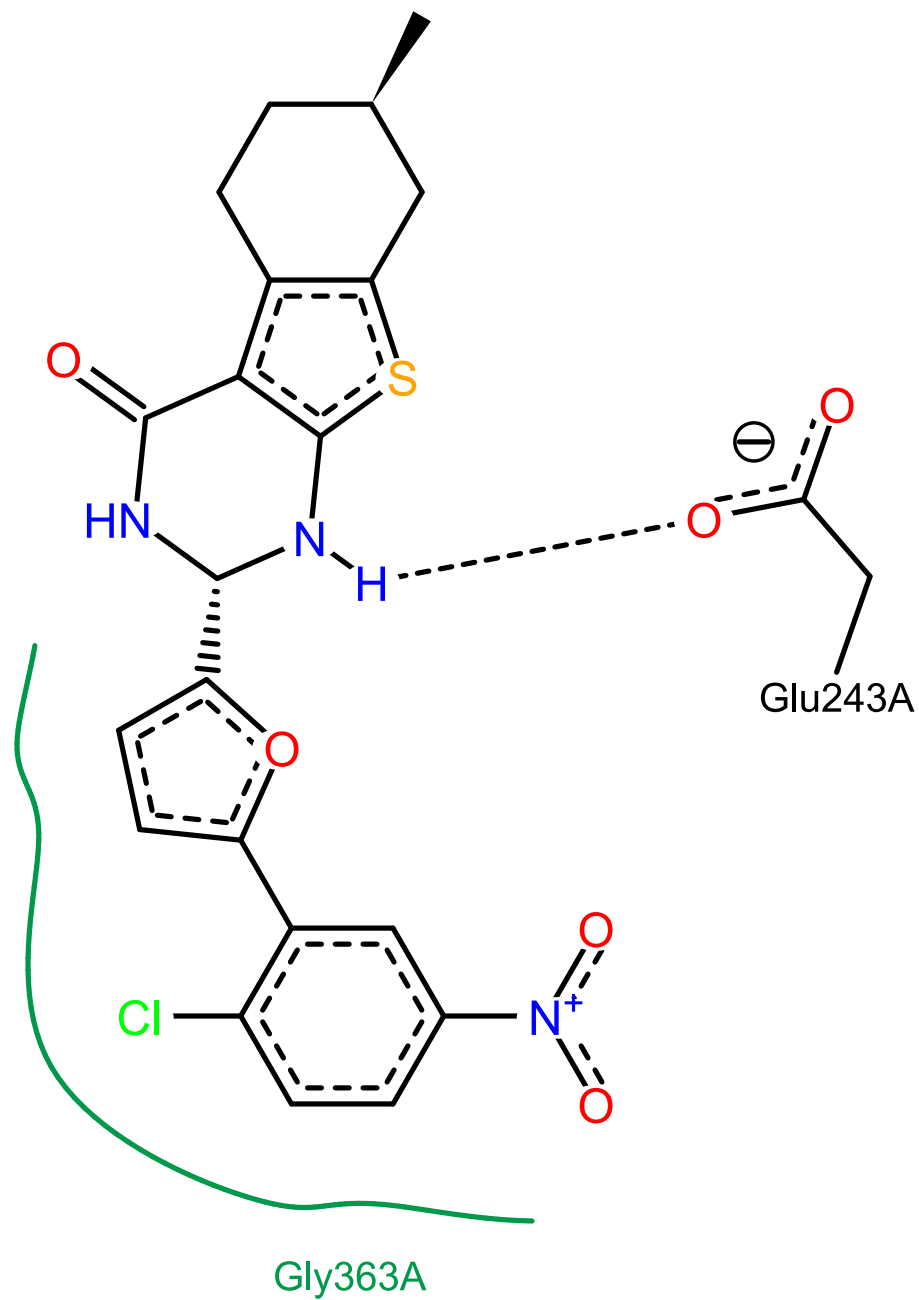

Supplement: S24 Fig — The dashed lines represent hydrogen bonds and the green spline segments illustrate hydrophobic contacts and green dots represent pi-pi stacking interaction. (PDF) [file pone.0207605.s024.pdf]

Ser45A

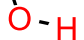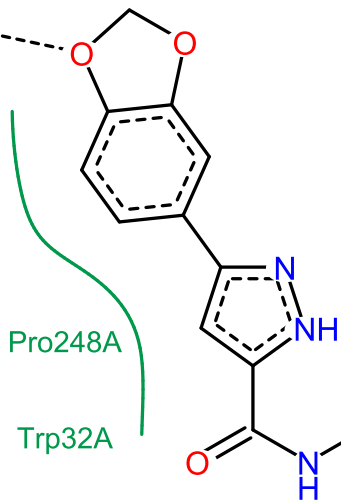

Ile165A

Leu33A

Met164A

Supplement: S25 Fig — The dashed lines represent hydrogen bonds and the green spline segments illustrate hydrophobic contacts and green dots represent pi-pi stacking interaction. (PDF) [file pone.0207605.s025.pdf]

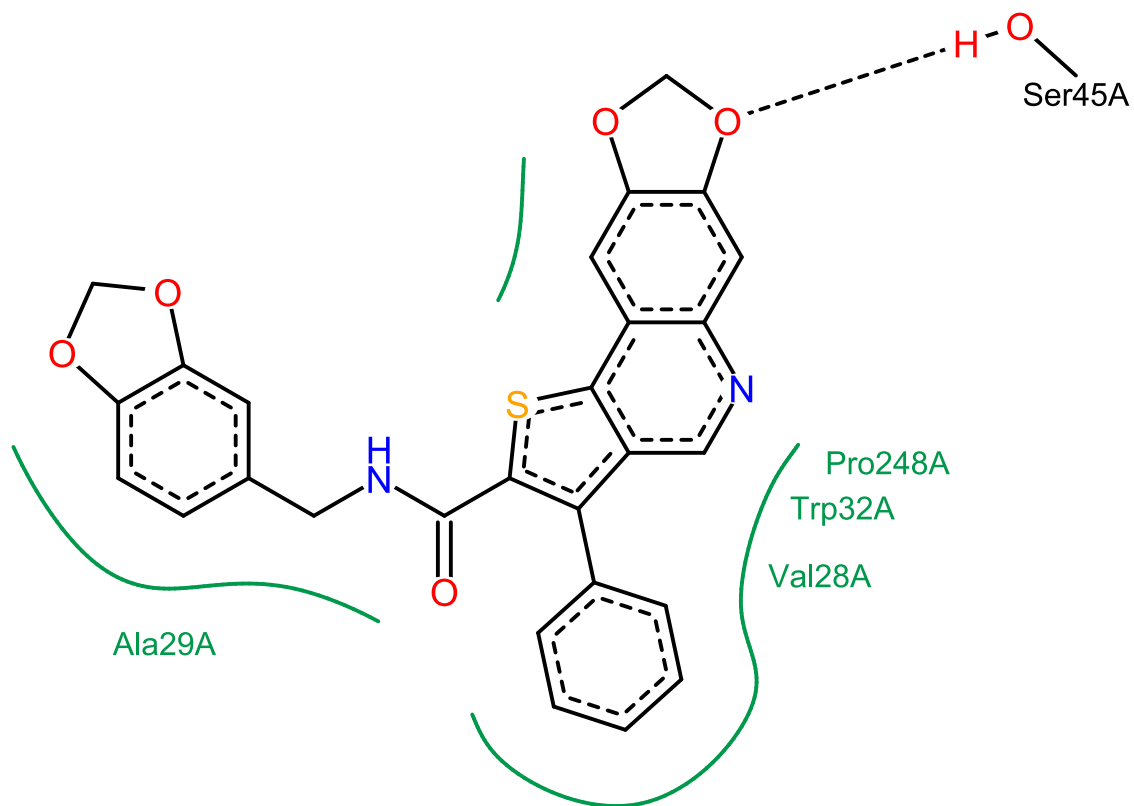

Supplement: S26 Fig — The dashed lines represent hydrogen bonds and the green spline segments illustrate hydrophobic contacts and green dots represent pi-pi stacking interaction. (PDF) [file pone.0207605.s026.pdf]

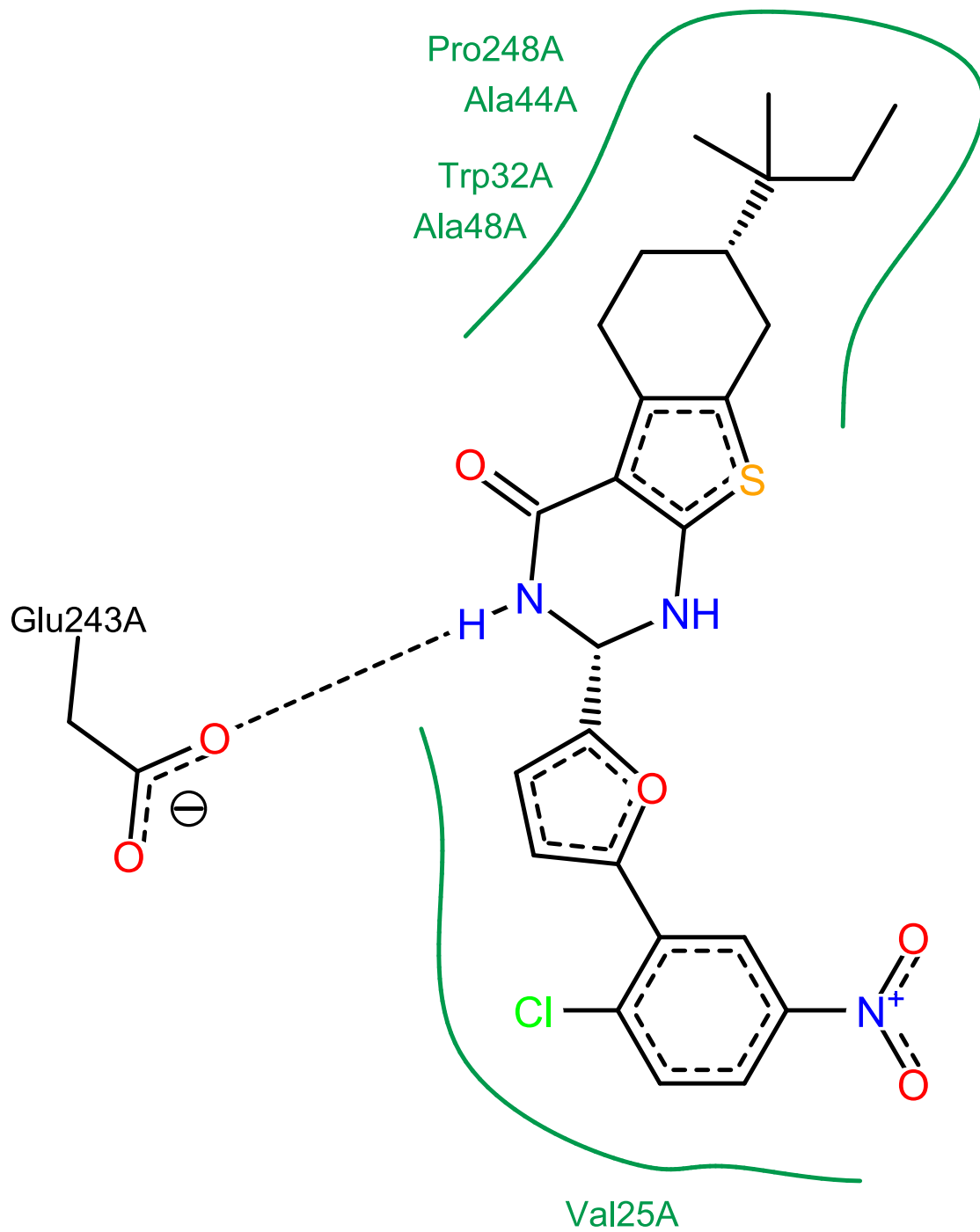

Supplement: S27 Fig — The dashed lines represent hydrogen bonds and the green spline segments illustrate hydrophobic contacts and green dots represent pi-pi stacking interaction. (PDF) [file pone.0207605.s027.pdf]

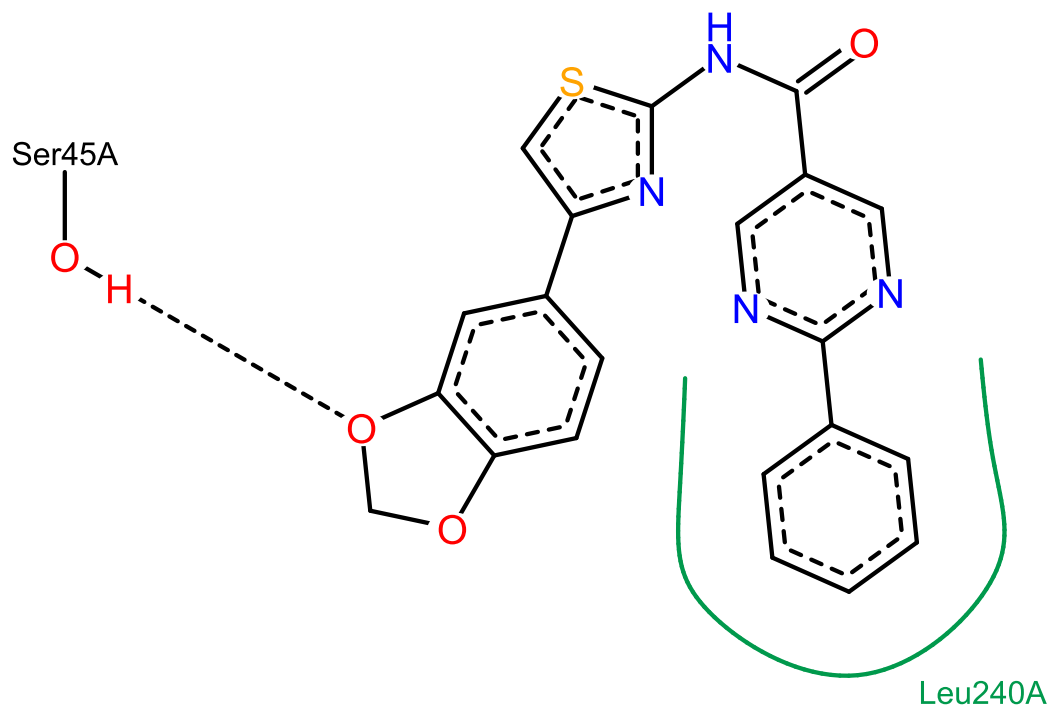

Supplement: S28 Fig — The dashed lines represent hydrogen bonds and the green spline segments illustrate hydrophobic contacts and green dots represent pi-pi stacking interaction. (PDF) [file pone.0207605.s028.pdf]

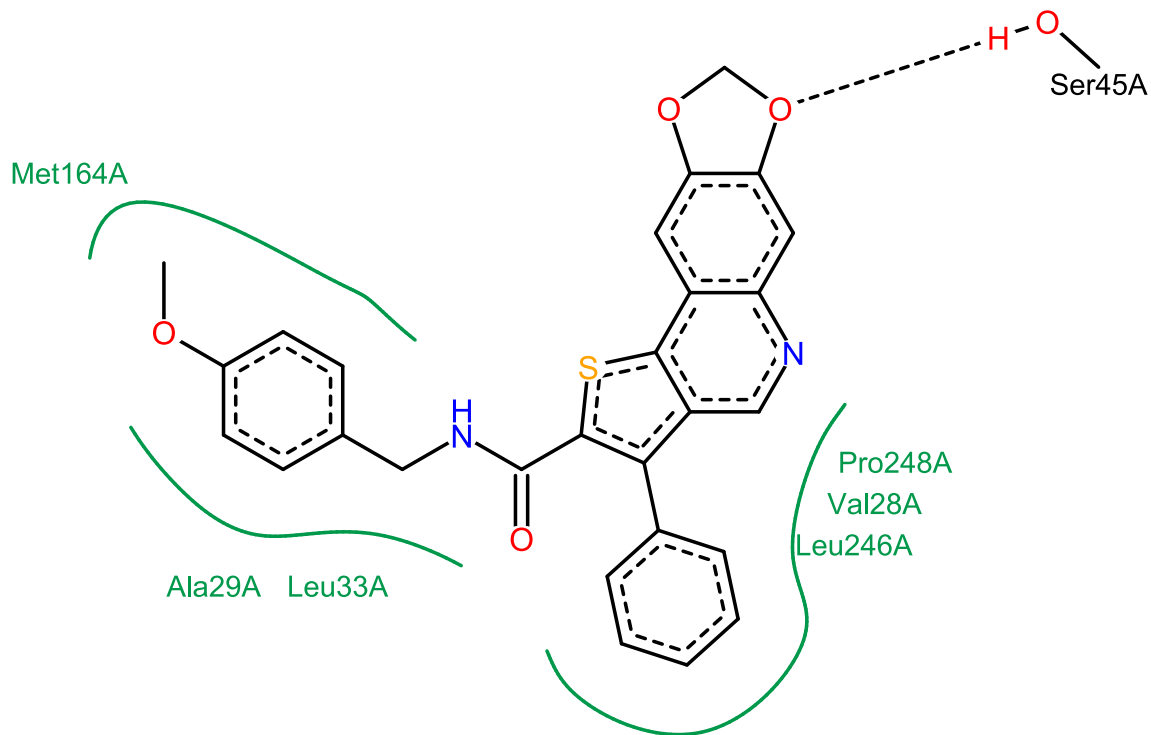

Supplement: S29 Fig — The dashed lines represent hydrogen bonds and the green spline segments illustrate hydrophobic contacts and green dots represent pi-pi stacking interaction. (PDF) [file pone.0207605.s029.pdf]

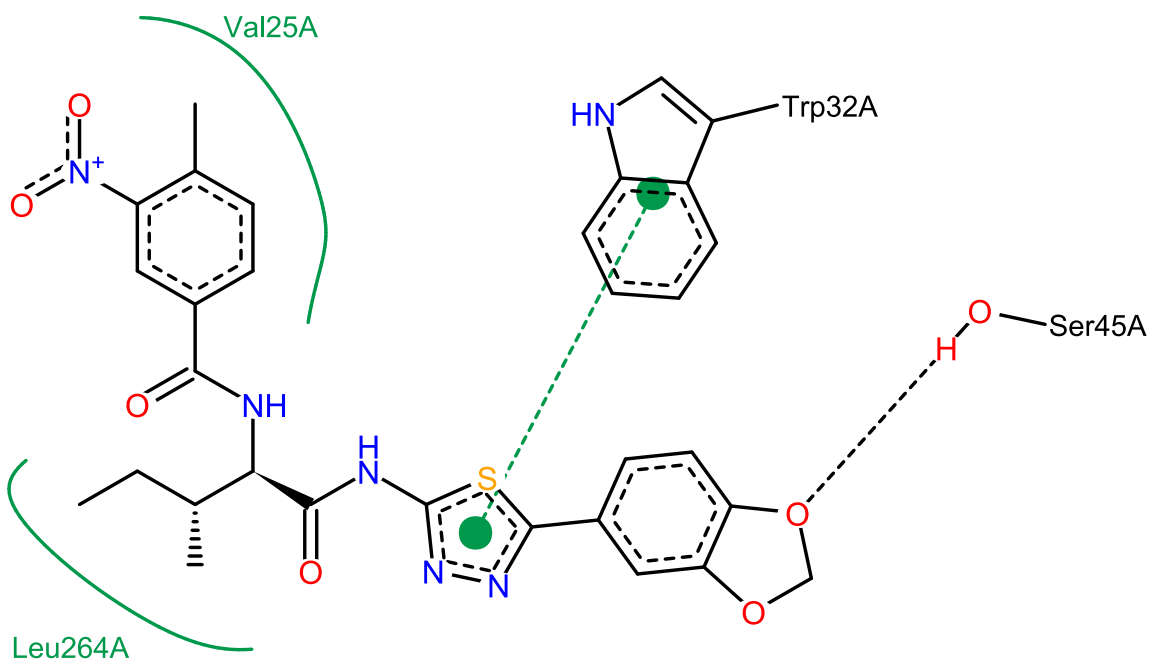

Supplement: S30 Fig — The dashed lines represent hydrogen bonds and the green spline segments illustrate hydrophobic contacts and green dots represent pi-pi stacking interaction. (PDF) [file pone.0207605.s030.pdf]

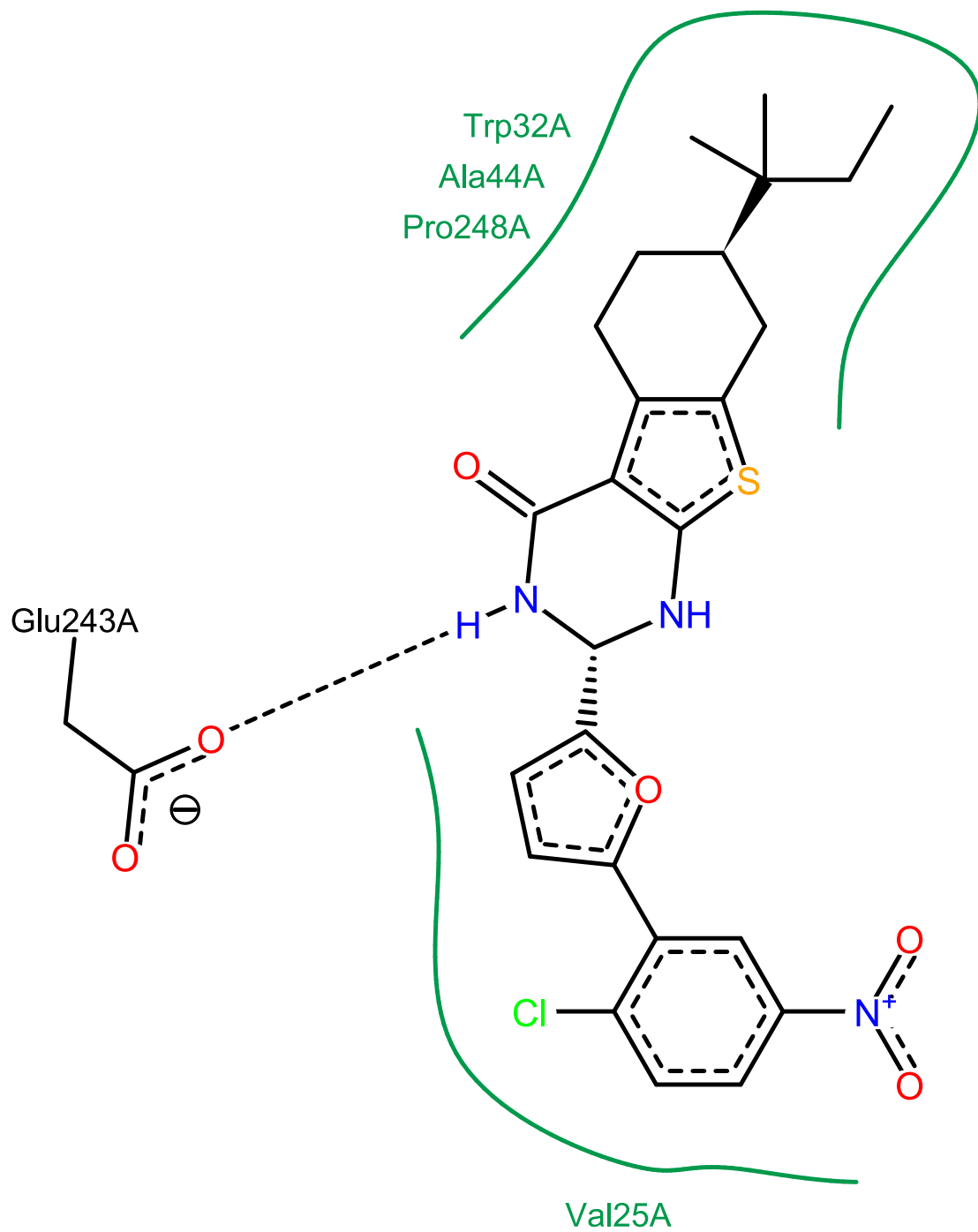

Supplement: S31 Fig — The dashed lines represent hydrogen bonds and the green spline segments illustrate hydrophobic contacts and green dots represent pi-pi stacking interaction. (PDF) [file pone.0207605.s031.pdf]

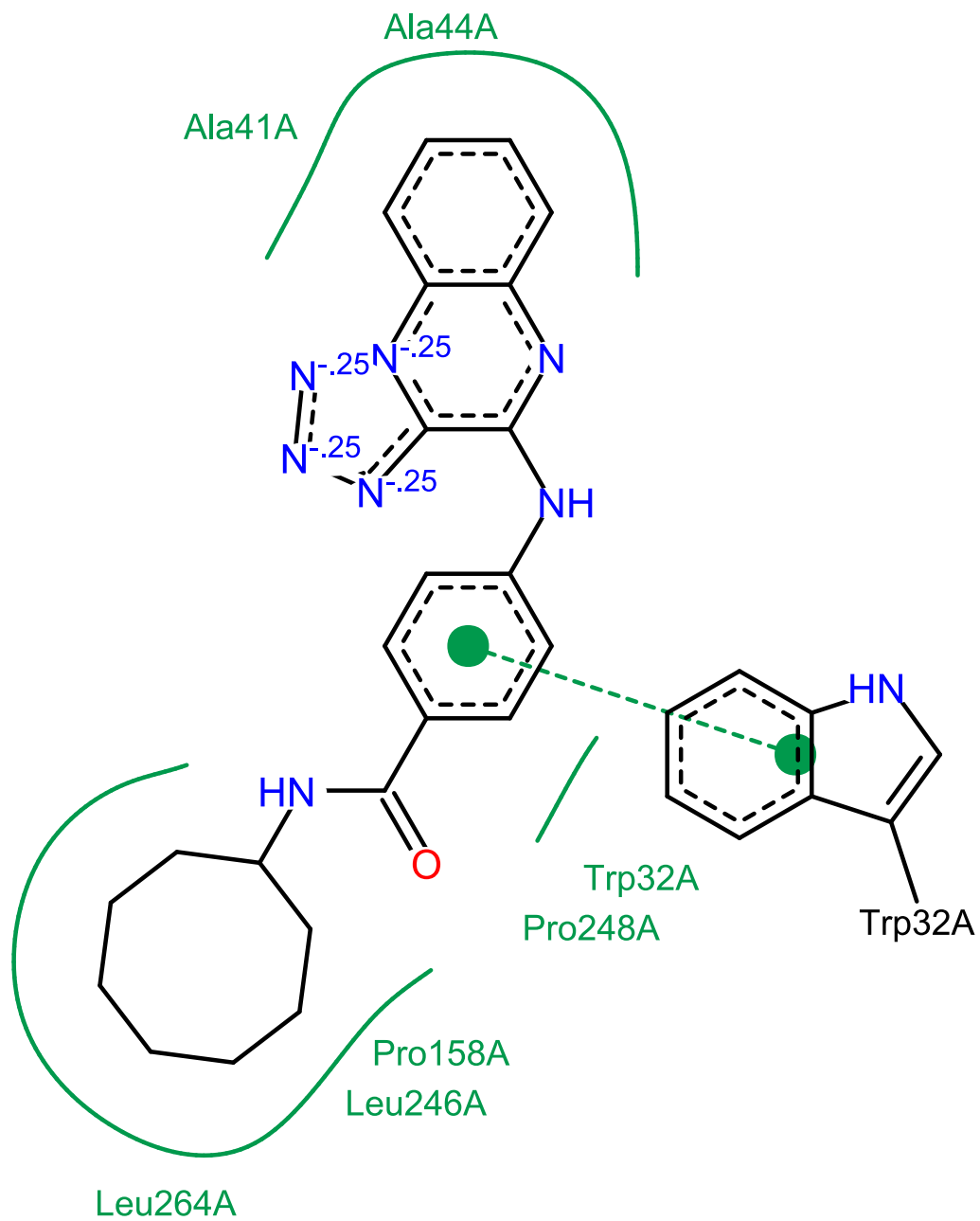

Supplement: S32 Fig — The dashed lines represent hydrogen bonds and the green spline segments illustrate hydrophobic contacts and green dots represent pi-pi stacking interaction. (PDF) [file pone.0207605.s032.pdf]

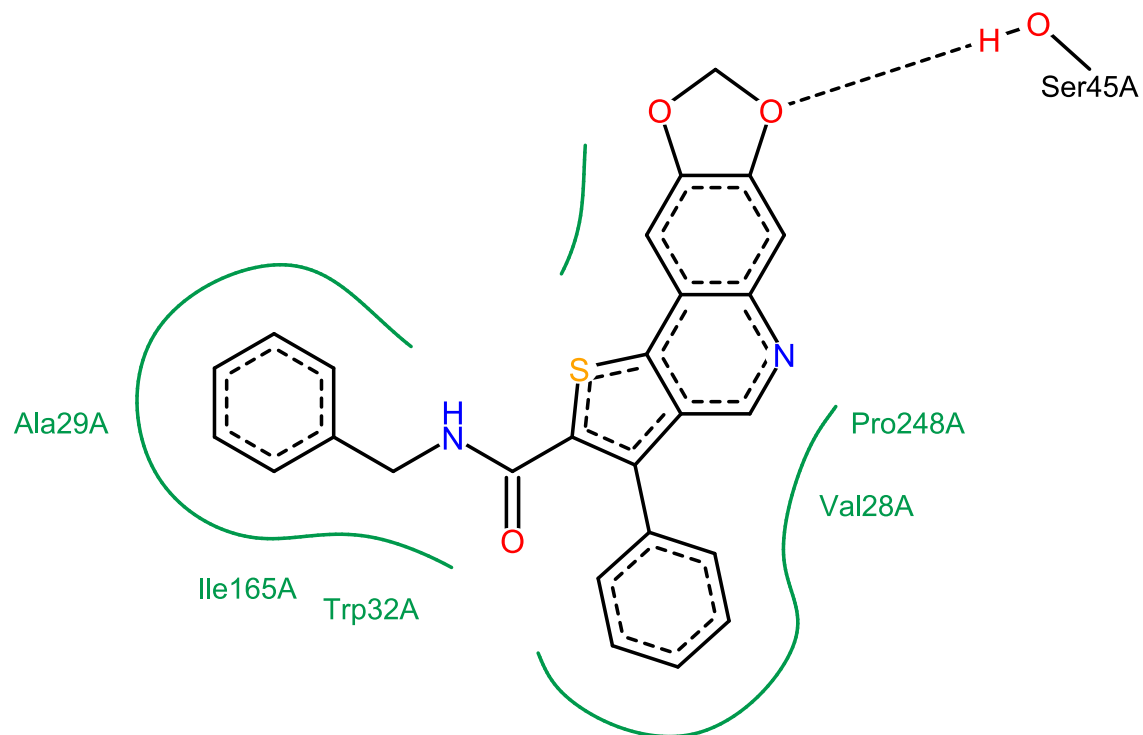

Supplement: S33 Fig — The dashed lines represent hydrogen bonds and the green spline segments illustrate hydrophobic contacts and green dots represent pi-pi stacking interaction. (PDF) [file pone.0207605.s033.pdf]

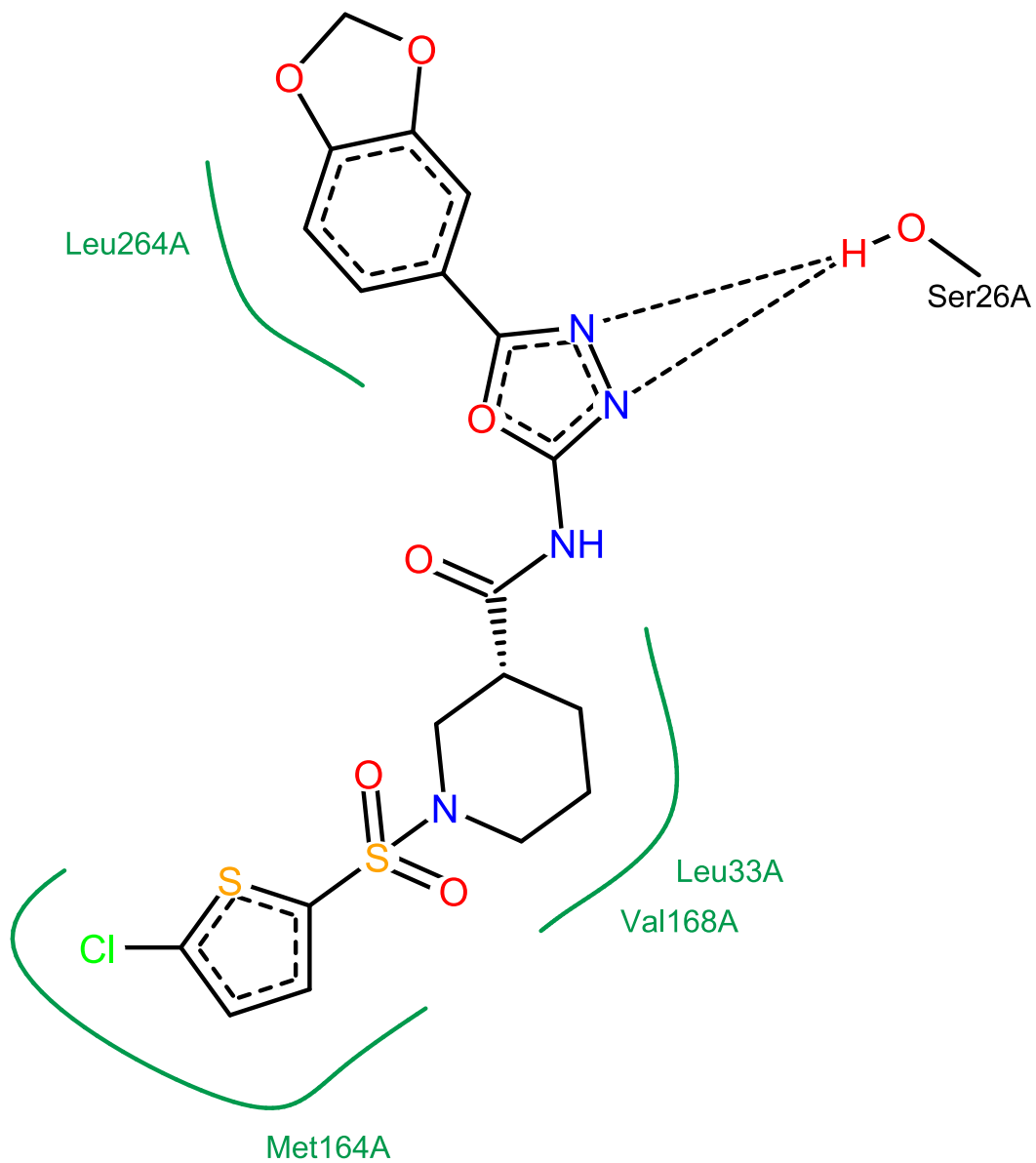

Supplement: S34 Fig — The dashed lines represent hydrogen bonds and the green spline segments illustrate hydrophobic contacts and green dots represent pi-pi stacking interaction. (PDF) [file pone.0207605.s034.pdf]

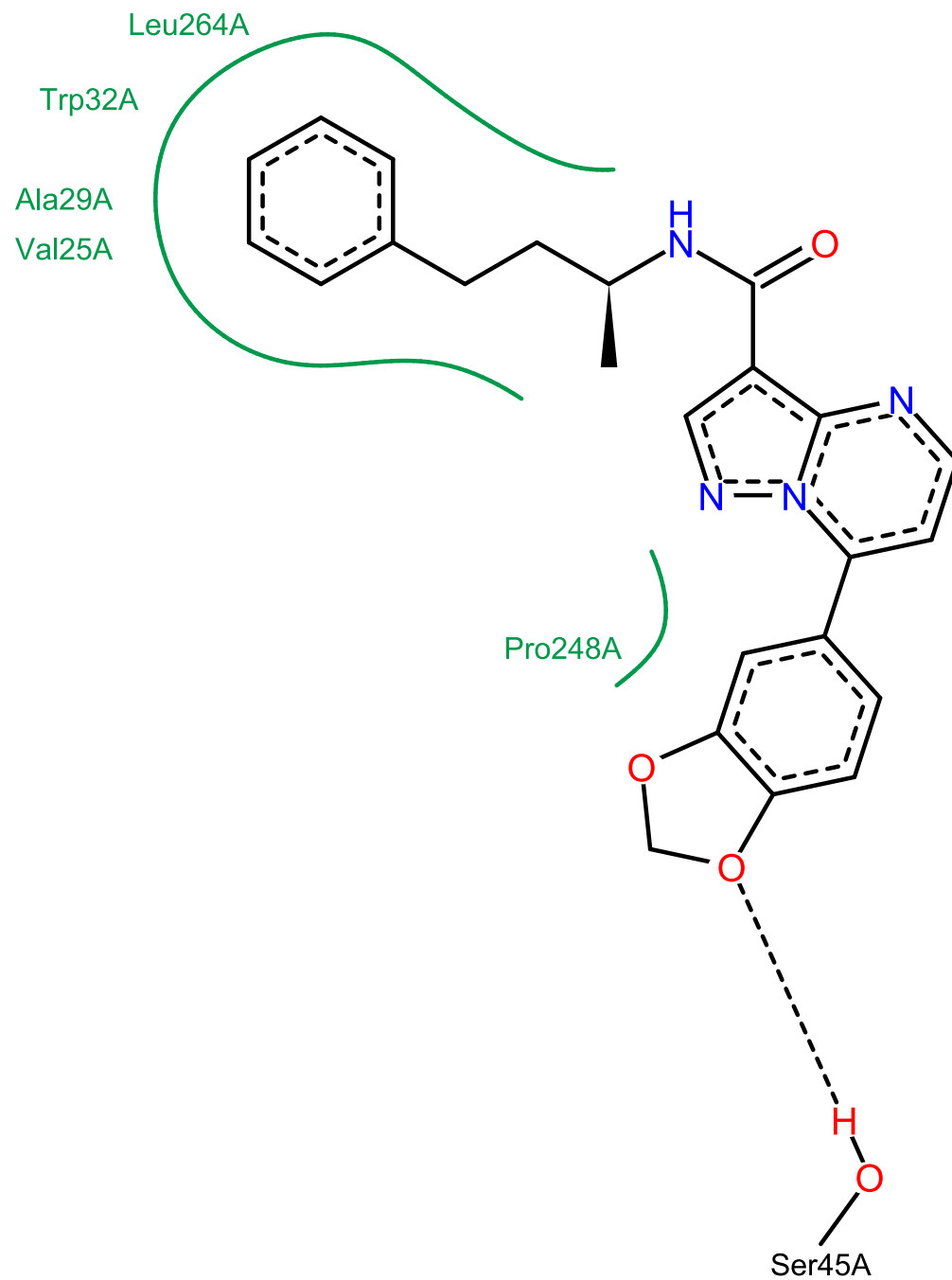

Supplement: S35 Fig — The dashed lines represent hydrogen bonds and the green spline segments illustrate hydrophobic contacts and green dots represent pi-pi stacking interaction. (PDF) [file pone.0207605.s035.pdf]

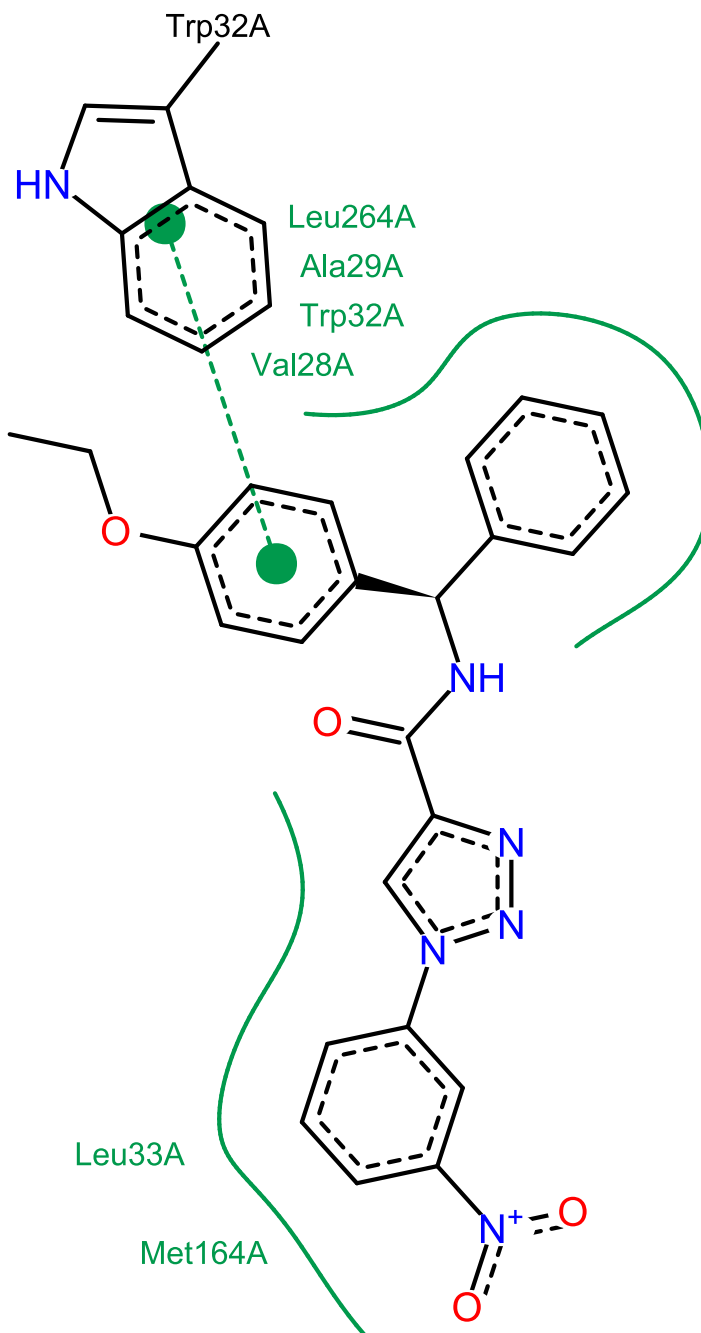

Supplement: S36 Fig — The dashed lines represent hydrogen bonds and the green spline segments illustrate hydrophobic contacts and green dots represent pi-pi stacking interaction. (PDF) [file pone.0207605.s036.pdf]

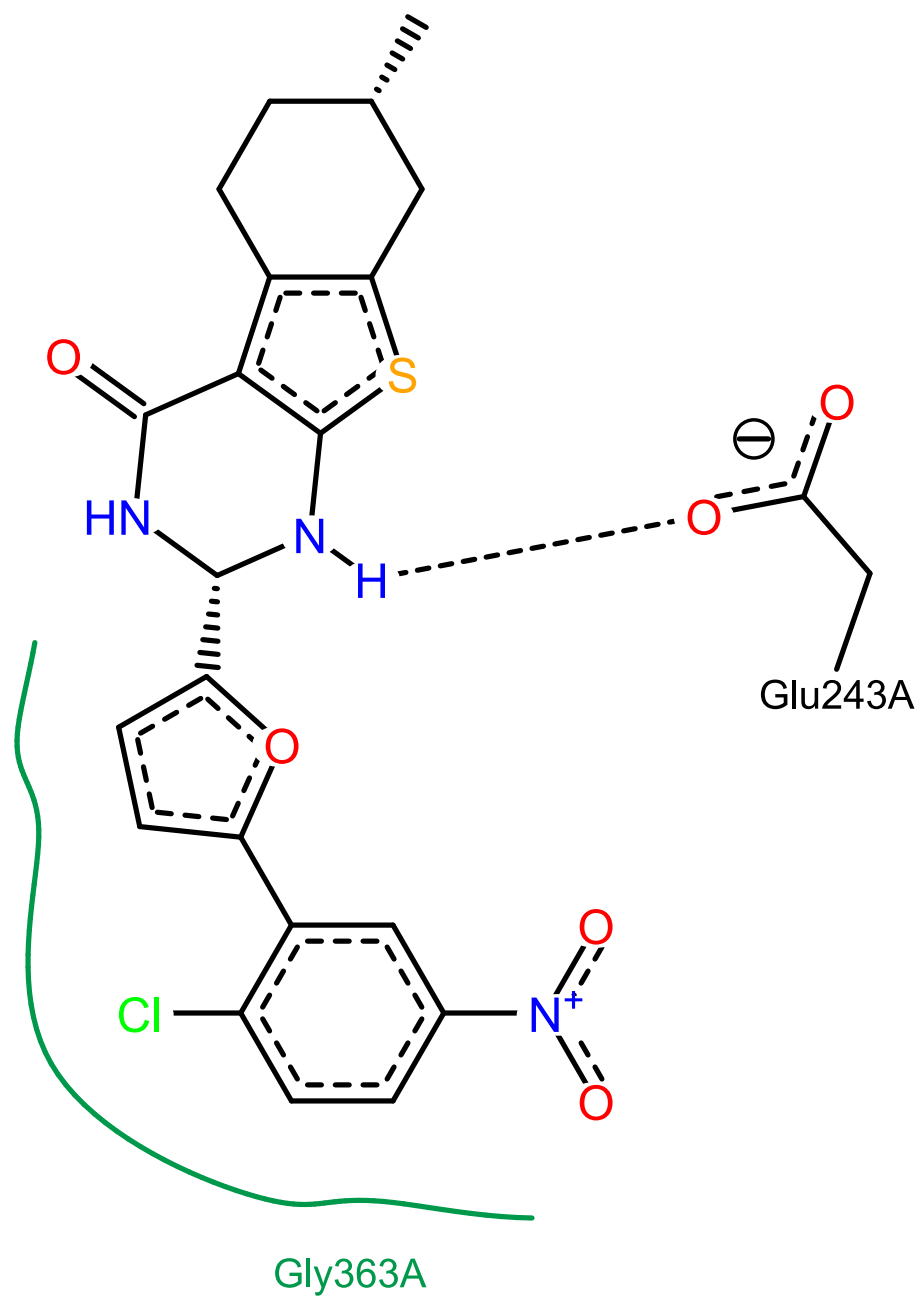

Supplement: S37 Fig — The dashed lines represent hydrogen bonds and the green spline segments illustrate hydrophobic contacts and green dots represent pi-pi stacking interaction. (PDF) [file pone.0207605.s037.pdf]

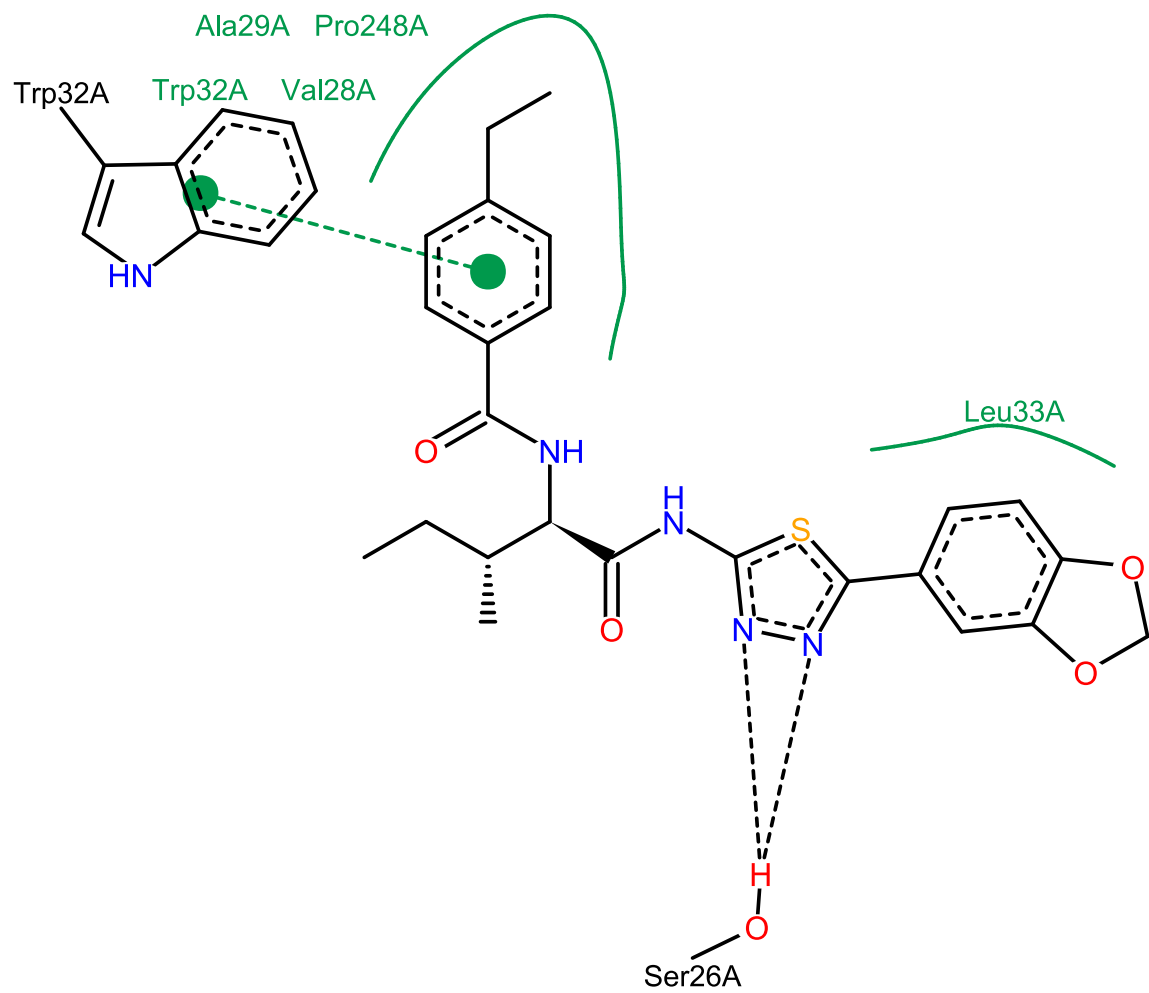

Supplement: S38 Fig — The dashed lines represent hydrogen bonds and the green spline segments illustrate hydrophobic contacts and green dots represent pi-pi stacking interaction. (PDF) [file pone.0207605.s038.pdf]

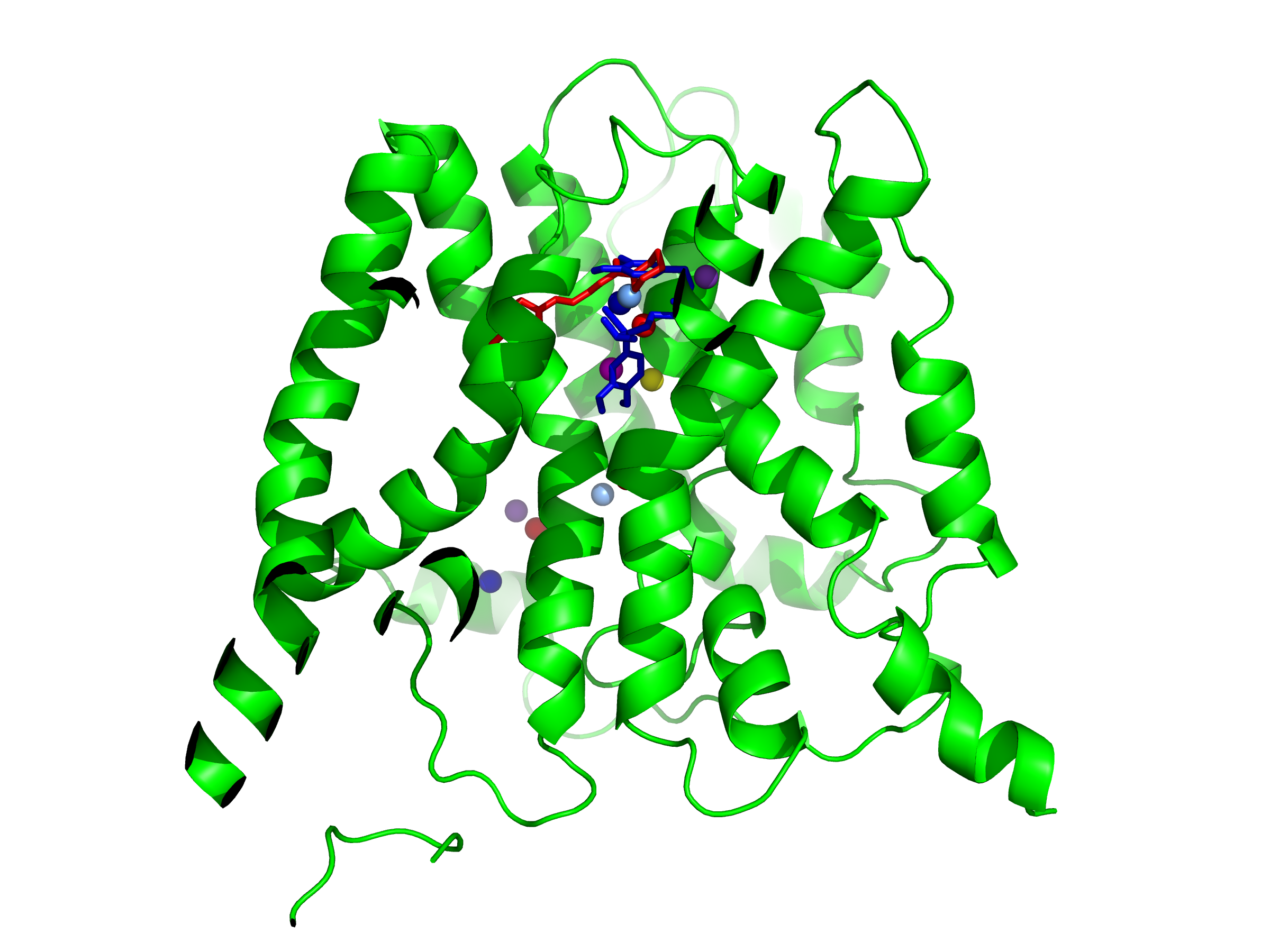

Supplement: S39 Fig — The different coloured spheres represent the different methods employed by Metapocket: LIGSITECS (purple), PASS (cyan), SURFNET (brown), Q-SiteFinder (blue), Fpocket (pink), ConCavity (orange), GHECOM (yellow) and POCASA (wheat) are all from their top 1 prediction where ligands piperine (red) and verapamil (blue) bind. The MetaPocket site for Rv1258c is shown as a red sphere. (PNG) [file pone.0207605.s039.png]
